# DENetwork unveils non-differentially expressed genes with functional relevance across conditions through information flow perturbation

**DOI:** 10.1093/nar/gkaf1350

**Published:** 2025-12-17

**Authors:** Bowen Zhao, Ting-Yi Su, Jingtao Wang, Quazi S Islam, Kailu Song, Steven K Huang, Matthieu Allez, Gregory J Fonseca, Carolyn J Baglole, Jun Ding

**Affiliations:** Department of Medicine, Division of Experimental Medicine, McGill University, Montreal H4A 3J1, Canada; Meakins-Christie Laboratories, Research Institute of the McGill University Health Centre, Montreal H4A 3J1, Canada; Quantitative Life Sciences, McGill University, Montreal H3A 1E3, Canada; Department of Medicine, Division of Experimental Medicine, McGill University, Montreal H4A 3J1, Canada; Meakins-Christie Laboratories, Research Institute of the McGill University Health Centre, Montreal H4A 3J1, Canada; Department of Pharmacology and Therapeutics, McGill University, Montreal H3G 1Y6, Canada; Meakins-Christie Laboratories, Research Institute of the McGill University Health Centre, Montreal H4A 3J1, Canada; Quantitative Life Sciences, McGill University, Montreal H3A 1E3, Canada; Division of Pulmonary and Critical Care Medicine, Department of Internal Medicine, University of Michigan, Ann Arbor 48109, United States; Division of Gastroenterology and Hepatology, Department of Medicine, McGill University Health Centre, Montreal H4A 3J1, Canada; Department of Medical Sciences, Khalifa University, Abu Dhabi 127788, United Arab Emirates; Meakins-Christie Laboratories, Research Institute of the McGill University Health Centre, Montreal H4A 3J1, Canada; Department of Pharmacology and Therapeutics, McGill University, Montreal H3G 1Y6, Canada; Department of Medicine, Division of Experimental Medicine, McGill University, Montreal H4A 3J1, Canada; Meakins-Christie Laboratories, Research Institute of the McGill University Health Centre, Montreal H4A 3J1, Canada; Quantitative Life Sciences, McGill University, Montreal H3A 1E3, Canada; School of Computer Science, McGill University, Montreal H3A 2A7, Canada; Mila-Quebec AI Institute, Montreal H2S 3H1, Canada

## Abstract

Differential gene expression (DE) analysis of RNA-sequencing (RNA-seq) data is a standard approach for identifying phenotypic differences between conditions. However, traditional DE methods such as DESeq2 focus on expression changes alone, often overlooking non-differentially expressed (non-DE) genes that may play key regulatory roles. This limits their ability to identify upstream drivers of transcriptomic variation. To address this gap, we introduce DENetwork, a network-based approach that prioritizes genes based on their influence on global information flow. Each gene is scored using an *in silico* knockout strategy that quantifies its impact across the inferred gene network, capturing both DE and non-DE genes with potential functional relevance. DENetwork deciphers intricate regulatory and signaling networks driving transcriptomic variations between conditions with distinct phenotypes. Across simulated and disease-relevant RNA-seq datasets, DENetwork identifies non-DE regulators enriched in known pathways and phenotypic associations, providing mechanistic insights missed by standard DE analysis, with implications for target discovery and intervention.

## Graphical Abstract

**Graphical Abstract:**
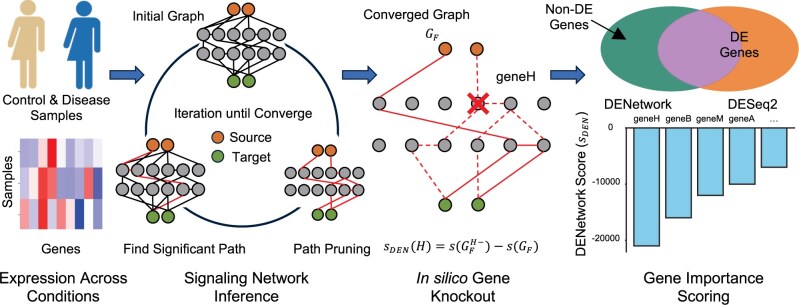

## Introduction

RNA sequencing (RNA-seq), as a high-throughput genomic technology, has been widely used to characterize biological state differences and global transcriptomic alterations. It has become a core approach for exploring molecular differences under diverse biological conditions. By comparing transcriptomes between healthy and diseased samples at a genome-wide scale, RNA-seq provides a solid foundation for the identification of biomarkers, understanding of disease mechanisms, and development of targeted therapies [1–3].

In RNA-seq data analysis, the most widely adopted strategy is the identification of differentially expressed (DE) genes, defined as those showing statistically significant expression changes between conditions. Methods such as DESeq2 [4] and edgeR [5] have been extensively applied in the study of complex diseases [6–11], significantly advancing our understanding of disease-associated molecular processes.

Despite their broad utility, these methods share a key limitation: they focus primarily on genes with significant transcriptional changes and tend to overlook those with stable expression levels that nonetheless play essential roles in regulatory networks. In many biological contexts, DE genes are often the consequences rather than the causes of cellular state changes. The true drivers underlying transcriptomic alterations are typically embedded in complex gene regulatory and signal transduction networks. Among the many overlooked regulators, a central component is the set of cellular receptors, which are specialized proteins located on the cell membrane or within the cell. These receptors sense external signals, such as proteins, peptides, hormones, or small molecules, by binding to specific ligands and initiate intracellular signaling cascades that ultimately modulate gene expression. Post-transcriptional regulators, such as RNA-binding proteins, further refine protein output and functional responses. This process of signal transduction determines how cells perceive and adapt to their environment and plays a central role in development, immunity, and disease progression. Therefore, if the research goal is to reverse abnormal cellular states (e.g., restoring diseased cells to a healthy-like state) through intervention, it becomes critically important to identify the upstream regulators in these signaling pathways, particularly receptors, signaling proteins, and transcription factors (TFs).

To overcome the limitations of conventional DE-based approaches, we developed a unified network inference framework called DENetwork. This method integrates RNA-seq expression data with prior biological knowledge of receptor-ligand interactions, signaling cascades, and TF–target gene regulation to construct condition-specific signaling networks. In this framework, receptors are assigned as source nodes and DE genes as target nodes. Building upon this structure, DENetwork implements an *in silico* knockout strategy that systematically evaluates the contribution of each gene to signal transduction. It then ranks all genes based on their impact on maintaining signal flow. This approach not only retains the ability of DE analysis to detect genes with strong expression shifts but also enables the identification of functionally important non-DE genes that occupy central positions in the network, particularly receptors and TFs that may serve as potential targets for therapeutic intervention.

We systematically evaluated the performance of DENetwork across multiple representative datasets, including infectious disease models (Influenza, SARS-CoV), genetic perturbation datasets (macrophage, HuR knockdown), and an endogenous disease model (liver cancer). Our results demonstrate that DENetwork outperforms traditional methods such as DESeq2 and network-based alternatives like SDREM in identifying known disease-associated genes. Importantly, the non-DE genes prioritized by DENetwork, which are often ignored in traditional analysis, exhibit strong biological relevance and predictive power in clinical contexts. These findings highlight the unique value of DENetwork in uncovering mechanistic regulators and expanding the interpretability of transcriptomic data, underscoring its broad potential for disease mechanism studies and target discovery.

## Materials and methods

### Data and data pre-processing

We utilized five bulk RNA-seq datasets spanning diverse biological contexts. Four of these datasets were publicly available. The first was derived from a study of human lung tissue infected with Influenza A virus (IAV), *Pseudomonas aeruginosa*, and *Mycobacterium bovis* (GSE192528); from this dataset, we selected four IAV-infected samples and five uninfected controls. The second dataset focused on SARS-CoV-2 infection in human lung tissue (GSE147507) [12] and included two infected samples and three uninfected controls. The third dataset included mouse macrophages from three wild-type and three *Alox15^−^*^/−^ samples. The fourth dataset consisted of liver cancer samples obtained from The Cancer Genome Atlas through the GDC portal, from which we randomly selected seven tumor samples and seven normal tissue samples. In addition to these publicly available datasets, we analyzed an in-house bulk RNA-seq dataset examining the effect of HuR (human antigen R) knockdown in human lung fibroblasts. This dataset included three control samples and three HuR-silenced samples.

The raw gene expression count matrix for each dataset was analyzed using DESeq2 [4] to identify DE genes between conditions. While DENetwork operates on DE gene lists as input, the identification of DE genes is not part of the DENetwork framework. Users are expected to derive their own DE gene sets using standard tools such as DESeq2 and can adjust statistical cutoffs according to their specific experimental goals and confidence requirements. DE genes were further stratified into upregulated and downregulated subsets, and depending on the biological context, either subset or both could be used as input to DENetwork. In this study, we primarily used a unified filtering criterion across datasets for finding DE genes: baseMean > 50, false discovery rate (FDR)-adjusted *P* <0.05, and an absolute log_2_ Fold-Change ($|\log _2(\mathrm{FC})|$) $> 0.6$ [4, 13, 14] to demonstrate the general applicability of DENetwork. Nevertheless, to illustrate the robustness of our framework under more stringent input conditions, we also applied a higher threshold of $|\log _2(\mathrm{FC})|$  $> 1.5$ for the HuR dataset. This adjustment was motivated by the observation that using the default 0.6 cutoff yielded an excessively large number (2032) of DE genes, which substantially increased the computational complexity of downstream signaling network inference. By applying a stricter cutoff, we focused the analysis on more confident and biologically meaningful transcriptional changes without affecting the core inference logic of DENetwork. This example illustrates the flexibility of our framework and its applicability across a range of DE gene selection strategies.

In addition to differential gene expression data, DENetwork requires a set of receptors specific to each dataset to initiate signaling flow simulations. For the Influenza dataset, we used 200 experimentally validated IAV receptors reported in the mt-STEM study [15]. For the SARS-CoV-2 dataset, we applied our previously published COVID2GeneList approach to identify 332 host receptors associated with viral entry and immune interactions [16]. For datasets without curated receptor lists, specifically the macrophage, HuR, and liver cancer datasets, we employed a unified and reproducible strategy: we retrieved all annotated receptors from the CellTalkDB database [17] and retained only those with detectable expression in the corresponding dataset. This approach ensured that the selected receptors were both biologically plausible and transcriptionally active in the system under study, thereby supporting reliable and context-aware network inference.

### The DENetwork pipeline

#### Building initial graph

A decisive feature of DENetwork is its ability to integrate condition-specific receptor inputs, DE genes, and a global protein interaction network to model upstream signaling mechanisms driving observed expression changes. Unlike conventional methods that rely solely on differential expression magnitude or pathway enrichment, DENetwork propagates signals through a biologically grounded interaction graph. This enables the identification of key regulators central to condition-specific signaling dynamics, including those with modest transcriptional changes.

**Figure 1.**
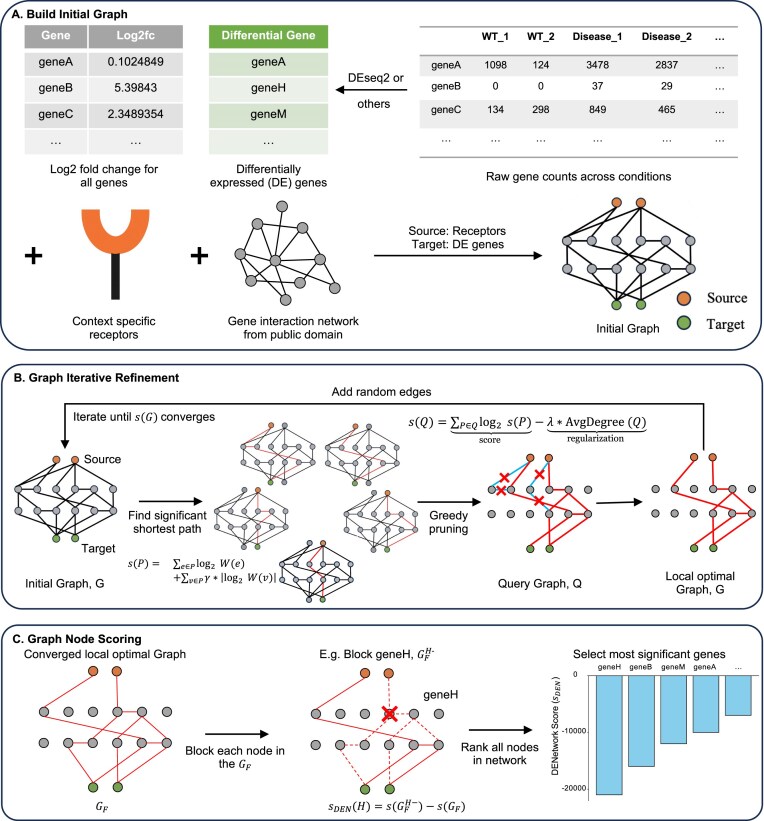
Flowchart of the DENetwork model. (**A**) Build initial graph: DENetwork begins by constructing a dataset-specific initial graph. Using context-specific receptor information and differential expression results (including DE genes and $\log _2(\mathrm{FC})$ from DESeq2), we extract a subgraph from a public gene interaction network that reflects genes relevant to the dataset. Receptor genes are then labeled as source nodes (orange) based on prior knowledge, DE genes or TFs (green) are labeled as target nodes, and other genes present in the dataset are treated as internal nodes (grey). (**B**) Graph iterative refinement: Starting from the initial graph, DENetwork enumerates all source–target paths with up to $K$ nodes. Each path is assigned a score $s(P)$, and a Gaussian distribution is fitted to the path score distribution to select statistically significant paths (5% cutoff). A greedy pruning strategy is then applied to iteratively remove paths with minimal contribution to the network score $s(Q)$, yielding a locally optimal graph. To escape local optima, random edges are added, and the refinement process is repeated until the network score $s(G)$ converges, resulting in a final optimized graph $G_F$. (**C**) Graph node scoring: To prioritize functional regulators, each node in the converged graph $G_F$ is systematically blocked, and the change in network score is computed as the DENetwork score. Nodes are ranked by the magnitude of score reduction, and those with the most significant impact are selected for downstream analysis.

To model signal transduction under specific biological conditions, we first constructed an initial gene interaction graph (Fig. 1A). This graph was constructed from high-confidence human protein–protein interaction (PPI) data in the HIPPIE database (version 2.0) [18]. We selected HIPPIE as the default resource because it prioritizes experimentally validated PPIs, offering higher reliability than STRING [19], which includes a substantial proportion of predicted interactions. In addition, our empirical evaluation on the liver cancer dataset (Supplementary Fig. S1) showed that HIPPIE produced prioritized gene sets with stronger enrichment of established cancer markers and achieved superior patient stratification in survival analysis compared to STRING. To ensure biological relevance, we retained only genes with detectable expression in the experimental dataset and included only PPI edges connecting these expressed genes. Edge weights were assigned using the normalized interaction confidence scores from HIPPIE, which range from 0 to 1. We then annotated the nodes within the network. Genes annotated as receptors and expressed in the dataset were designated as source nodes, representing upstream signaling initiators. Genes identified as DE by DESeq2 were labeled as target nodes. If a gene met both criteria, it was classified as a source node to preserve its upstream functional role. Each node was also assigned an attribute corresponding to its $|\log _2(\mathrm{FC})|$ between conditions, providing a quantitative measure of transcriptional change that is later used in signal propagation. To address the concern that incorporating $\log _2(\mathrm{FC})$ might bias DENetwork toward prioritizing genes with larger expression changes, we examined the distribution of $\log _2(\mathrm{FC})$ values among prioritized genes (Supplementary Fig. S2A). We observed that many top-ranked genes exhibited relatively modest expression differences ($\log _2(\mathrm{FC})$  $<$ 0.6), indicating that DENetwork does not rely solely on transcriptional magnitude. Rather, final prioritization is determined by the amount of signal flow passing through each node (Supplementary Fig. S2B). This design enables the identification of functionally important genes, even those with limited expression changes, provided they serve as key conduits in condition-specific signaling pathways.

DENetwork also supports the use of TFs as target nodes for constructing the initial interaction network. To identify TFs whose target genes are significantly enriched for DE genes, we used TF–target interaction data provided by the iDREM software [20] and applied hypergeometric enrichment testing. For each candidate TF, we counted the total number of its known target genes ($n$) and the number of those that were DE genes ($x$). Let $N$ denote the total number of genes in the dataset and $K$ the number of DE genes. The enrichment *P* was calculated using the cumulative hypergeometric distribution: $P = \sum _{k = x}^{n} \frac{\binom{K}{k} \binom{N - K}{\, n - k\, }}{\binom{N}{n}}$, where $C(a, b)$ denotes the binomial coefficient $\binom{a}{b}$. Since TF target genes often have higher baseline expression levels than background genes, they may exhibit greater statistical power to be detected as DE genes, even in the absence of true biological enrichment. To assess this potential confounder, we examined the Influenza dataset and compared the average expression levels (based on raw counts) between all genes and the subset of TF target genes (Supplementary Fig. S3). A Kolmogorov–Smirnov test yielded a statistic of 0.137, suggesting that although the associated *P* is extremely small (due to the large sample size), the overall distributional difference is minor. Following multiple testing corrections using either the FDR or Bonferroni method, TFs with adjusted *P* < 0.05 were included in the final network as target TF nodes. In this study, we used the NetworkX Python package [21] to construct and manage the condition-specific gene interaction network, leveraging its graph data structures and algorithms to support signal flow modeling in DENetwork.

#### Finding significant paths from sources to targets

To identify biologically relevant signaling routes, we enumerated all possible source–target paths between receptor nodes (sources $S$) and DE nodes (targets $T$) within the gene interaction network. To ensure biological plausibility and control computational complexity, we imposed a constraint on the maximum number of nodes per path, denoted as $K$. The default value of $K$ was set to 5, based on both the SDREM [22] network construction strategy and our own empirical optimization (Supplementary Fig. S4A), which indicated that allowing up to five nodes in a path provided better performance than higher values. Importantly, we verified that path scores were not biased by path length: score distributions across 1-hop to 5-hop paths were comparable (Supplementary Fig. S5), confirming that DENetwork does not inherently favor shorter or longer paths. Formally, the path enumeration function $X(S, T, k)$ is defined as:


(1) \begin{eqnarray*}
X(S, T, k) = \arg \min _{P \in Q_k} \sum _{e \in P} 1, \quad k \le K
\end{eqnarray*}

where $Q_k$ represents the set of all valid paths consisting of $k$ nodes. In this step, all edges were treated as unweighted (weight = 1) to identify the minimal-length paths.

Each candidate path $P$ was then assigned a score $s(P)$ that integrates both topological confidence and expression dynamics:


(2) \begin{eqnarray*}
s(P) = \sum _{e \in P} \log _2 W(e) + \sum _{v \in P} \gamma \cdot |\log _2 W(v)|
\end{eqnarray*}

Here, $W(e)$ is the interaction score for edge $e$, and $W(v)$ is the fold change of node $v$. The parameter $\gamma$ modulates the relative influence of node-level transcriptional changes versus edge-level network topology. Log_2 _transformation was applied to reduce skewness and avoid score saturation. By default, we set $\gamma = 1.0$, based on empirical tuning results (Supplementary Fig. S4B). Notably, both increasing and decreasing $\gamma$ from this value led to reduced model performance, indicating that a balanced weighting of node and edge information is optimal for DENetwork.

After obtaining the path scores, we assessed their statistical significance by assuming that the path scores follow a Gaussian distribution, which is supported by empirical distributions (Supplementary Fig. S6), and calculated a *P* for each path score as follows:


(3) \begin{eqnarray*}
P(s) = 1 - 0.5 \left[ 1 + \text{erf} \left( \frac{s - \mu }{\sigma \sqrt{2}} \right) \right]
\end{eqnarray*}

where $s$ is the path score, and $\mu$ and $\sigma$ are the empirical mean and standard deviation of all scores. Paths with *P* < 0.01 were considered statistically significant and retained. This threshold was selected based on benchmarking results (Supplementary Fig. S4C), and adopting a more permissive *P* threshold resulted in reduced model performance.

To further refine the network structure, we reduced redundancy and promoted sparsity by selecting the top $N$ most probable paths for each $(S, T)$ pair, as defined by the following scoring function:


(4) \begin{eqnarray*}
M_p(S, T) & = & {\rm arg\, max} _{P \in Q} \prod _{e \in P} \mathrm{Pr}(e) \\
&=& \arg \max _{P \in Q} \sum _{e \in P} \left[ \log _2 W(e)\right. \\
&&\left. + |\log _2 W(se)| + |\log _2 W(te)| \right]
\end{eqnarray*}

In this formulation, $M_p(S, T)$ denotes the set of top-ranked paths based on the estimated likelihood of signal propagation. The probability of each edge $e$ is defined such that its log-transformed score corresponds to the sum of the log-transformed PPI score $W(e)$ and the $|\log _2(\mathrm{FC})|$ of its source ($se$) and target ($te$) nodes. These multiplicative components are log-transformed to enable additive scoring. The default value of $N$ was set to 5 (see Supplementary Fig. S4D), as we observed that retaining a larger number of paths led to unstable performance of DENetwork.

#### Searching for a locally optimal network

To construct a concise yet informative signaling subnetwork, we applied a greedy backward-pruning strategy that iteratively removes the lowest-scoring paths. At each step, the path with the lowest score (as defined by Equation 2) was eliminated, and a new graph $Q$ was generated. The quality of the resulting network was evaluated using a regularized scoring function:


(5) \begin{eqnarray*}
s(Q) = \underbrace{\sum _{P \in Q} \log _2 s(P)}_\text{score} - \underbrace{\lambda * \text{AvgDegree}(Q)}_{\text{ regularization}}
\end{eqnarray*}

Equation (5) consists of two components: a score term that quantifies the overall informativeness of the network, defined as the sum of the log-transformed scores of all retained paths in graph $Q$, and a regularization term that penalizes network complexity based on the average node degree. To balance the contributions of these two terms, the regularization coefficient $\lambda$ was set by default to the ratio between the mean of the score term and the mean of the average degree across internal networks.

At each step of the pruning process, we removed the lowest-scoring path, updated the network $Q$, and computed the corresponding regularized score $s(Q)$. As expected, $s(Q)$ generally declined as more paths were eliminated, reflecting a trade-off between signal retention and structural sparsity. We plotted $s(Q)$ against the number of paths removed (Supplementary Fig. S7) and selected the network corresponding to the “elbow” or “knee” of the curve. This graph was considered the locally optimal network, balancing biological relevance and interpretability.

#### Iteratively refining the local-optimal network

To escape potential suboptimal solutions produced by greedy pruning, we implemented an iterative refinement procedure to improve the initially selected network $ G$ and identify a more optimal configuration $ G_F$ (Fig. 1B and Supplementary Fig. S8, which provides a more detailed overview of the model’s iterative refinement process). In each iteration, we randomly added a small number of candidate edges to the current network, and then re-ran the core inference workflow to re-optimize the network structure.

Each refinement iteration included the following key steps: (i) building a full graph using all original nodes and the updated edge set, (ii) identifying significant shortest paths between source and target nodes, (iii) applying the greedy backward-pruning strategy to search for a locally optimal subnetwork based on the regularized score (Equation 5), and (iv) evaluating whether the resulting network represented an improvement over the previous iteration.

The iterative process continued until convergence, as determined by two hyperparameters: *v*, the minimum relative improvement in network score required per iteration (set to 5%), and *t*, the maximum number of consecutive iterations permitted without substantial improvement (set to 5). Specifically, refinement terminated when the network score increased by less than v% for *t* successive iterations. These default values were selected based on empirical tuning (Supplementary Fig. S4E and F). Notably, setting a smaller improvement threshold (*v*) resulted in incomplete model optimization, whereas increasing *t* beyond this value did not provide any substantial additional benefit. The complete graph refinement procedure is presented as pseudocode in Algorithm 1.

**Algorithm 1.** Finding a local-optimal network.

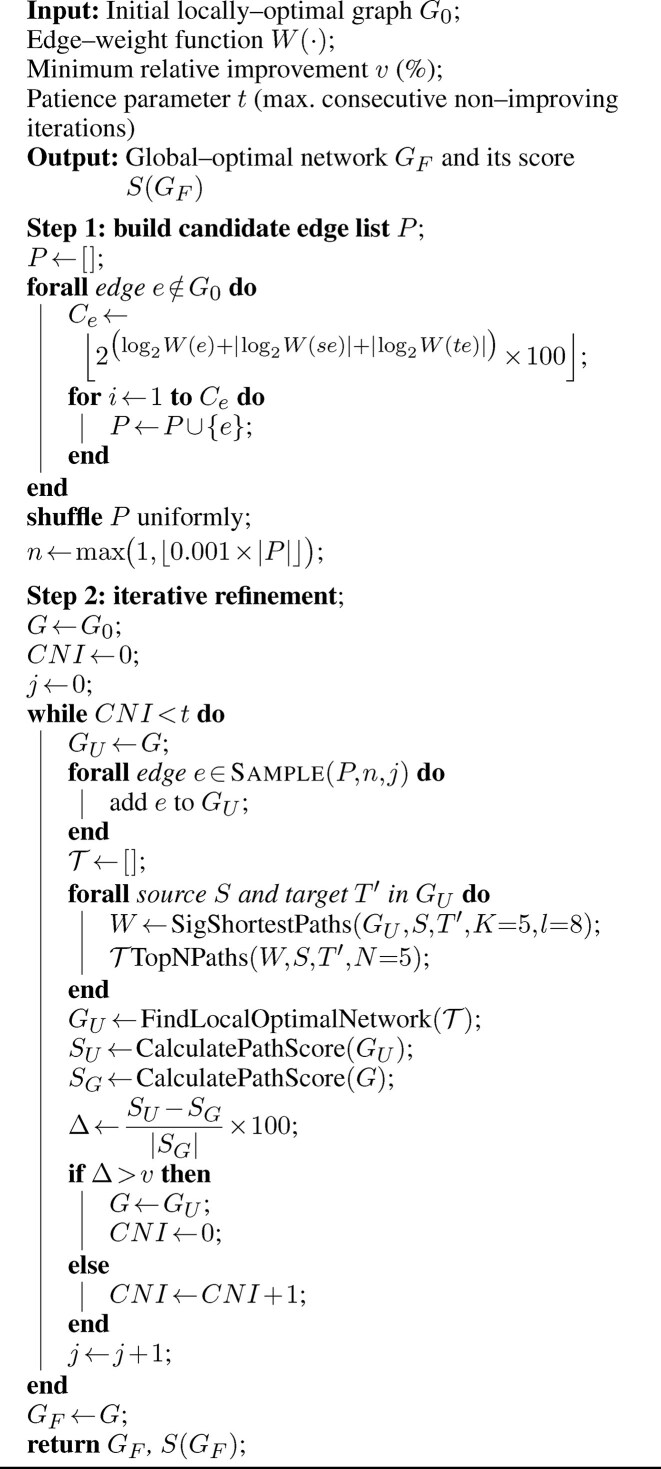

### Node scoring and ranking

To evaluate the functional importance of each gene in the final network, we applied an *in silico* node knockout procedure, analogous to a virtual gene knockout, on the converged locally optimal graph $ G_F$. For each node (gene) $ H$, we constructed a perturbed version of the network, denoted $ G_F^{H-}$, by removing the node and all of its incident edges. This simulates the removal of the gene’s regulatory and signaling influence on the network.

We then recalculated the DENetwork score ($s_{\text{DEN}}$) for the perturbed network and defined the impact score for gene $H$ as the change in the global network score resulting from its *in silico* knockout: $s_{\text{DEN}}(H) = s(G_F^{H-}) - s(G_F)$, where $ s(G)$ is the regularized network score defined in Equation (5), incorporating both pathway strength and sparsity penalties. Because the same scoring function is used consistently, no re-tuning is required during knockout simulations. Genes with the most negative $ s_{\text{DEN}}$ values (i.e., those whose removal causes the greatest drop in overall network score) are ranked highest, as their presence is critical for maintaining effective signal propagation in the condition-specific regulatory network. This approach enables DENetwork to detect upstream regulators with functional importance that may not be apparent from transcriptional changes alone.

To ensure robustness, we evaluated whether the rankings were biased toward either highly connected or highly expressed genes. As shown in Supplementary Fig. S2A and B, the top-ranked genes included both hub nodes and low-degree nodes, indicating that prioritization was not driven solely by network centrality or expression amplitude. We further examined the relationship between node degree and knockout-based importance scores (Supplementary Fig. S9) and observed only a weak correlation, confirming that DENetwork rankings capture a gene’s functional contribution to information flow rather than degree alone. The resulting ranked gene list thus provides a mechanistically informed prioritization of condition-specific regulators, suitable for downstream applications such as biomarker discovery, gene set enrichment analysis (GSEA), or experimental validation.

### Systematic evaluation

#### Visualizing DENetwork results

We visualized the top 100 genes ranked by DENetwork scores using Cytoscape, removing disconnected nodes to enhance interpretability. Nodes were styled by type: source nodes (orange), internal nodes (gray), and target nodes (green). Node size reflected the magnitude of the DENetwork score, with larger nodes indicating higher inferred regulatory importance. To further illustrate expression dynamics, we generated heatmaps for top-ranked DE and non-differentially expressed (non-DE) genes across conditions. Literature references were used to validate the biological relevance of selected predictions.

#### Gene set enrichment analysis

To assess functional relevance, we performed GSEA using the PreRanked module from the GSEA toolkit [23], with the Reactome pathway database as the reference. Genes were ranked by DENetwork based on their contribution to the global network score (i.e., DENetwork score), and by DESeq2 based on FDR-adjusted *P*. For each ranked list, we ran GSEAPreRanked and visualized the most significantly enriched pathways.

#### Pathway enrichment via hypergeometric testing

We conducted hypergeometric enrichment analysis across multiple datasets to evaluate pathway-level significance. Gene sets included: (i) all DENetwork-prioritized genes, (ii) the non-DE subset from DENetwork, (iii) DESeq2-identified genes, and (iv) the union of DENetwork and DESeq2 genes. For each group, we extracted the top 10 enriched Reactome pathways and assessed their disease relevance, providing a comparative measure of each method’s ability to recover context-specific biological processes.

#### Benchmarking against other graph-based methods

We systematically compared DENetwork with SDREM and DESeq2 by evaluating the overlap of identified genes with curated disease-associated gene sets via hypergeometric enrichment. To isolate the contribution of non-DE genes, we repeated the analysis after excluding all DE genes. Additionally, we compared the biological coherence of union gene sets from DENetwork + DESeq2 versus SDREM + DESeq2 using Gene Ontology Biological Process (GOBP) enrichment. This enabled a functional-level comparison between network-based inference strategies.

#### Survival analysis

To assess the clinical relevance of gene sets, we performed survival analysis on the liver cancer dataset using TCGA bulk RNA-seq data. Patients were stratified into high- and low-risk groups according to the expression of gene sets derived from DENetwork (full and non-DE subset), SDREM, and DESeq2. Kaplan–Meier survival curves were compared using the two-sided log-rank test, and *P* values were reported to quantify prognostic performance.

#### Scalability assessment

We evaluated the scalability of DENetwork by measuring runtime and memory usage across five representative datasets (Supplementary Fig. S10). Results showed a clear logarithmic trend with respect to dataset size, demonstrating that DENetwork is computationally efficient and suitable for large-scale analyses.

### Simulation validates the robustness of DENetwork in identifying regulatory factors

To comprehensively evaluate the robustness of DENetwork in identifying key regulatory factors, we designed a two-part simulation study. The primary goal was to assess whether DENetwork could accurately recover signal transduction cascades under both ideal conditions and realistic noise disturbances. By systematically testing the method in these contrasting scenarios, we aimed to verify its reliability in prioritizing causal regulators.

First, we constructed a clean synthetic dataset based on the Influenza interactome, which includes 172 receptors, 296 TFs, and 15,322 genes. To preserve realistic network topology, we randomly selected 5% of receptors (8 of 172) and traced all paths of length $\le 3$ from these receptors to TFs and subsequently to genes. The top 5% most connected TFs (14) and downstream genes (760) were retained, forming an implanted signaling cascade consisting of 782 nodes (8 receptors + 14 TFs + 760 genes). This sparsity level reflects the modular structure of real signaling networks while ensuring sufficient propagation for signal detection. Expression values of genes within the cascade were doubled ($\log _2(\mathrm{FC})$ = 1.0), while the rest of the genes remained unchanged.

Applying DENetwork to this synthetic dataset, we found that all eight implanted receptors and 13 of the 14 TFs ranked within the global top-100 list. In total, 32 receptors and 32 TFs were identified among the top-ranked genes, yielding F_1_ scores of 0.40 for receptors (precision = 0.25, recall = 1.00) and 0.565 for TFs (precision = 0.406, recall = 0.929) (Supplementary Fig. S11). These results indicate that DENetwork effectively captures causal nodes with high recall and minimal false negatives. While the precision is moderate, especially for receptors, this reflects a trade-off inherent in DENetwork’s design, which emphasizes comprehensive signal recovery over strict sparsity. In practical settings, such broad upstream inference is often desirable for exploratory discovery.

To further evaluate robustness under realistic conditions, we introduced expression noise into the real Influenza dataset. Specifically, we applied Gaussian perturbations to 5%, 10%, or 20% of genes in 10 independent replicates per noise level. Additionally, we simulated dropout noise by setting the same proportions of log_2_FC values to zero, generating a total of 60 noisy datasets. Despite these disturbances, DENetwork rankings remained remarkably stable: the overlap between each noisy run and the original top-100 list ranged from 82% to 84% under Gaussian noise and 80% to 88% under dropout noise (Supplementary Fig. S12). These consistently high overlap rates demonstrate that DENetwork rankings are robust to both stochastic fluctuations and missing signals, preserving key regulatory signals even under noisy or degraded input conditions.

### Hyperparameter choices and statistical cutoffs in DENetwork analysis

To ensure the reliability and interpretability of DENetwork results, we selected a unified set of default hyperparameters and statistical thresholds based on biological prior knowledge. These biologically informed defaults were then shown to be generally applicable across diverse datasets, supporting their use as standard settings in our analyses. Specifically, we conducted a systematic evaluation of multiple parameter combinations on five representative datasets, including Influenza, SARS-CoV-2, Macrophages, HuR knockdown, and Liver Cancer. Each parameter configuration was independently tested five times to assess robustness and reproducibility. In each run, DENetwork was used to construct a local signaling network under the given settings, from which the resulting gene set was subjected to Gene Ontology (GO) enrichment analysis. GO terms that were consistently enriched across all runs were extracted, and their statistical significance (*P*) was used as a performance metric. The results from each parameter setting across all datasets were aggregated and visualized using boxplots of $-\log _{10}(P)$, with the median indicated by an orange line (Supplementary Fig. S4). This evaluation confirmed that the selected default parameters robustly identified functionally enriched genes across different biological conditions. Based on these results, we selected the following optimal hyperparameters for all subsequent analyses: $K = 5$ in Equation (1), $\gamma = 1$ in Equation (2), $p(s) = 0.01$ in Equation (3), $N = 5$ in Equation (4), a minimum improvement threshold $v = 5\%$, and a stopping criterion of $t = 5$ consecutive iterations without improvement.

Furthermore, when not relying on biological priors, DENetwork provides an automated strategy for optimal parameter selection based on the score of the final locally optimal graph s(Q) (Equation 5), which integrates the sum of optimized path scores with a regularization term. To support this, we implemented a cross-validation interface (see our GitHub page), enabling systematic hyperparameter tuning when desired.

Moreover, to systematically identify statistically significant key genes from DENetwork ranking results, we adopted a permutation-based statistical inference framework. Specifically, for each dataset, we repeatedly randomized the edges of the PPI network while preserving both the node number and degree distribution. DENetwork was then run on each permuted network, resulting in the accumulation of over 100,000 background scores that collectively define a robust empirical null distribution for gene scores. Based on this background distribution, we set the significance threshold at the empirical score corresponding to a *P* of 0.05. For each gene in the real network, if its DENetwork score exceeded this threshold, it was considered a high-confidence regulatory candidate. This strategy eliminates the need for subjective cutoffs, is fully grounded in statistical inference, and, through large-scale permutation testing, effectively controls the false positive rate and enhances the reliability and scientific rigor of key gene identification.

### HuR small interfering RNA (siRNA) knockdown experiments

#### Derivation and culture of primary human lung fibroblasts

Human lung fibroblasts were derived from lung tissue obtained from the University of Michigan Lung Biorepository through donor lungs provided by Gift of Life, Michigan [24] and cultured in Gibco™ Minimum Essential Media (MEM) (Thermo Fisher Scientific, USA) containing 10% fetal bovine serum (FBS; Hyclone Laboratories, Logan, UT) supplemented with gentamicin (WISENT Inc, Canada), Antibiotic-Antimycotic (WISENT Inc, Canada), and GlutaMAX (Thermo Fisher Scientific, USA). Cells were maintained at 37°C in a humidified 5% CO_2_–95% air incubator and were between passages 7 and 10. A total of six samples were prepared for RNA-seq, including three control and three HuR knockdown fibroblast samples.

#### HuR siRNA

Fibroblasts were seeded into $T_{25}$ cell culture flasks containing 4 ml of 10% FBS/MEM and allowed to grow overnight for 24 h. Transfections were performed with either 60 nM of HuR small interfering RNA or control (scrambled) siRNA (siCtrl; Santa Cruz, CA). siRNA-transfected cells were incubated for an additional 24 h and harvested.

#### RNA sequencing

Total RNA was extracted from human lung fibroblasts using the Trizol (Bio-Rad) as per the manufacturer’s protocol. Quantification of RNA was done using Qubit (Thermo Scientific), and its quality was assessed using the 2100 Bioanalyzer (Agilent Technologies). Transcriptome libraries were generated using the KAPA RNA HyperPrep (Roche) with poly-A selection (Thermo Scientific). The starting material was 250 ng of total RNA. The library preparation steps included poly-A selection, RNA fragmentation, first and second-strand cDNA synthesis, A-tailing, adapter ligation, and library amplification. Adapters used were xGen Dual Index UMI Adapters (IDT) with unique barcodes. Equimolar amounts of libraries were pooled and sequenced on the Illumina NextSeq 500 platform, obtaining ~20 M single-end 75 bp reads per sample. RNA-seq was conducted at the Institute for Research in Immunology and Cancer of the University of Montreal. Data quality was assessed with FastQC [25] and MultiQC [26]. Alignment was performed using STAR 2.7.8a [27], and non-uniquely mapped reads were discarded with Samtools 1.12 [28]. Picard Tools 2.23.3 [29] were used for alignment quality assessment and PCR duplication rate analysis. Gene expression was quantified using featureCounts [30] from Subread 2.0.1, and DESeq2 [31] was employed for differential expression analysis as indicated earlier.

## Results

### DENetwork reveals critical regulators orchestrating infectious disease

Infectious diseases often induce dramatic transcriptomic remodeling and alterations of the tissue microenvironment by activating host immune responses and signaling pathways. Deciphering the regulatory factors involved in the infection process is essential for understanding disease mechanisms and developing targeted therapies. To systematically investigate the host regulatory mechanisms underlying IAV infection, we applied DENetwork to an RNA-seq dataset comprising healthy controls and IAV-infected individuals. Receptor genes known to interact with IAV (Supplementary Fig. S13 shows that the selected receptor genes are significantly associated with the curated influenza disease gene set, $P = 7.1 \times 10^{-12}$) were designated as source nodes, and the upregulated DE genes identified by DESeq2 were used as target nodes.

**Figure 2.**
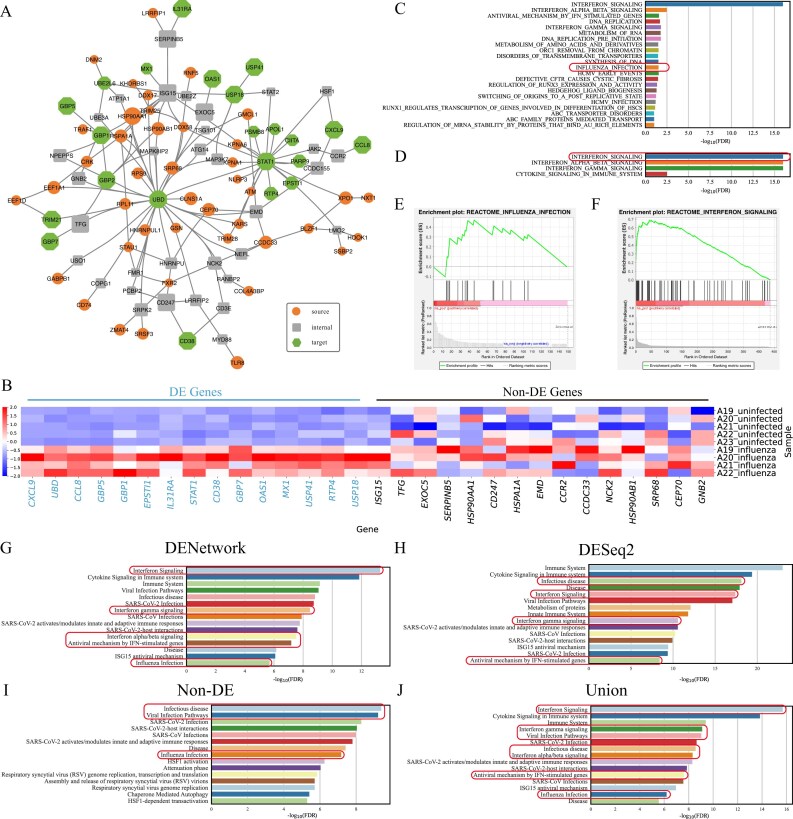
DENetwork uncovers influenza-associated genes. (**A**) Network graph visualizing the top 100 genes within the final DENetwork-derived network. Nodes not contributing to network connections were excluded. Source nodes (receptors), internal nodes, and target nodes (upregulated DE genes) are represented by ellipses, rectangles, and hexagons, respectively. A larger node size signifies a higher impact (ranking) in the network. (**B**) Heatmap showing the expression profiles of 15 upregulated DE genes and 15 non-DE genes across uninfected (uninfected_*) and Influenza-infected (infected_*) samples. Upregulated genes are highlighted in blue; non-DE genes are marked in black. (**C, D**) GSEAPreRanked analysis results. (C) uses the DENetwork-based ranking (descending node scores), while (D) uses the DESeq2-based ranking (ascending *P*). Representative pathways highly relevant to the Influenza dataset are highlighted with boxes. GSEA enrichment plots of representative pathways identified by DENetwork ranking (**E**) and DESeq2 ranking (**F**). Reactome pathway enrichment analysis performed on four distinct gene sets: all genes in the DENetwork-derived network (**G**), upregulated DE genes identified by DESeq2 (**H**), non-DE genes within this network (**I**), and the union of DENetwork and DESeq2 gene sets (**J**). Each panel displays the top 15 most significant pathways, with influenza-associated pathways highlighted by boxes.

Based on the above input, DENetwork reconstructed an IAV-associated signaling network consisting of 425 genes that passed statistical filtering and were retained as putative key regulators (all results were obtained using a consistent set of statistical thresholds and hyperparameter settings). For effective visualization and interpretation, we focused on a representative subnetwork comprising the top 100 genes ranked by network importance scores. This approach maintains biological relevance while improving readability, as rendering the full 425-gene network would obscure key insights due to excessive visual complexity. Among the top 100 genes, three were disconnected from the main component and were excluded. The resulting final visualized network (Fig. 2A) thus contained 97 connected genes, including 42 source nodes (known receptors, 26 of which were DE but were labeled as source nodes rather than target nodes, while the remaining 16 were non-DE), 22 target nodes (upregulated DE genes), and 33 internal signaling molecules (all non-DE genes). These node categories were distinguished by shape and color, and node size reflected their relative network ranking, with larger nodes indicating higher inferred importance. The complete DENetwork-derived signaling network, including all 425 prioritized genes, is provided in Supplementary Table S1 for comprehensive reference and reproducibility. Top-ranked subnetworks are often used to highlight central regulators [32], while the complete network is made available for exhaustive evaluation.

Among the top-ranked genes, we observed a significant enrichment of upregulated DE genes identified by DESeq2 (hypergeometric enrichment $P = 7.8 \times 10^{-30}$), which are known to participate in host antiviral responses to IAV infection, such as *CXCL9, UBD, CCL8, GBP5, GBP1, EPSTI1, IL31RA, STAT1, GBP7, OAS1, MX1, USP41, RTP4*, and *USP18* (Fig. 2B, left). Most of these genes are well-established interferon-stimulated genes (ISGs) or chemokines that play pivotal roles in early immune responses. For instance, *CXCL9* and *CCL8* promote immune cell recruitment to infected sites [33, 34]; *STAT1* serves as a master regulator of interferon signaling [35]; *MX1* and *OAS1* are antiviral effectors [36]; and *USP18* functions as a negative regulator to fine-tune interferon signaling [37, 38].

Beyond the DE genes above, DENetwork also identified non-DE genes that were not detected by conventional expression-based methods but are functionally relevant in the context of IAV infection (Fig. 2B, right). These include *ISG15, TFG, EXOC5, SERPINB5, HSP90AA1, CD247, HSPA1A, EMD, CCR2, CCDC33, NCK2, HSP90AB1, SRP68, CEP70*, and *GNB2*. While *ISG15* did not exhibit significant changes in expression in this dataset, it has been well-documented as an interferon-induced antiviral gene during influenza infection [39]. *CCR2* plays a key role in monocyte recruitment and lung inflammation in response to IAV [40]. Members of the heat shock protein family such as *HSP90AA1* and *HSP90AB1* may support viral replication by modulating protein folding and stability during infection [41].

The discovery of these non-DE but functionally critical genes highlights the unique advantage of DENetwork. Traditional differential expression analyses primarily focus on expression magnitude changes between conditions, which may miss regulators that are functionally essential but transcriptionally stable. In contrast, DENetwork does not prioritize genes based on expression differences alone. Instead, it models the signaling flow from receptor source nodes to DE target genes and assigns importance scores to internal nodes based on their necessity in mediating this flow. Genes that serve as critical conduits or bottlenecks in these signal propagation paths are prioritized, even if their expression levels do not change significantly. This approach enables DENetwork to uncover structurally and functionally essential regulators that play key roles in the signaling cascade, providing a more mechanistic, network-based view of host responses to infection. To ensure that these genes were truly non-DE, rather than false negatives resulting from limited statistical power, we performed two additional analyses. First, we relaxed the $\log _2(\mathrm{FC})$ threshold used in the differential expression analysis (Supplementary Fig. S14). Second, we applied the two-sided Wilcoxon rank-sum test, a non-parametric alternative to DESeq2, to enhance sensitivity in detecting expression differences (Supplementary Fig. S15). In both cases, the selected non-DE genes remained statistically non-significant, indicating that their classification was not due to overly stringent thresholds or underpowered statistical tests. These results further support the robustness and distinctiveness of DENetwork in identifying mechanistically important regulators overlooked by conventional methods, thereby providing a more comprehensive, systems-level perspective on the host response to IAV infection.

To further validate the biological relevance of genes prioritized by DENetwork, we applied two complementary functional enrichment strategies: preranked GSEA, which evaluates the global biological coherence of the full DENetwork-derived gene ranking, and hypergeometric GSEA, which tests the functional relevance of top-ranking gene subsets identified by DENetwork.

GSEA using DENetwork-ranked genes (Fig. 2C) revealed strong enrichment for influenza-specific pathways, including “Influenza Infection” *$(\mathit {P} = 3.1 \times 10^{-2})$* and “Antiviral mechanism by IFN-stimulated genes” *$(\mathit {P} = 3.2 \times 10^{-2}),$* suggesting that the network-derived gene ranking captures regulators specifically involved in IAV responses. In contrast, GSEA based on DESeq2 *P* rankings (Fig. 2D) primarily enriched for broader immune processes such as “Interferon signaling” *$(\mathit {P} = 5.0 \times 10^{-17})$* and “Cytokine signaling in immune system” *$(\mathit {P} = 3.1 \times 10^{-3}),$* with little evidence of IAV-specific regulation. This distinction was clearly illustrated in the enrichment plots (Fig. 2E and F), where DENetwork displayed a sharp leading-edge enrichment for influenza-related pathways, while DESeq2 results reflected more general immune activity.

Similarly, Reactome pathway enrichment analysis confirmed that DENetwork prioritizes functionally relevant genes (Fig. 2G–J). Notably, the DENetwork-identified non-DE genes (Fig. 2I) retained strong enrichment for virus-specific pathways even after excluding all DESeq2-significant genes, highlighting their structural centrality and biological relevance. In contrast, DESeq2-identified genes alone (Fig. 2H) were predominantly linked to general immune or stress-related responses and failed to capture influenza-specific signatures. Importantly, when DENetwork and DESeq2 gene sets were combined (Fig. 2J), the enrichment for influenza-related pathways was substantially enhanced reflecting the complementary strengths of the two approaches. This union, consisting of both DENetwork-derived non-DE genes and DESeq2-significant DE genes (non-DE + DE), yielded a more comprehensive and biologically coherent view of the host response than either method alone. We also provide DENetwork results using enriched TFs as target nodes for reference (see Supplementary Fig. S16).

To evaluate whether DENetwork’s ability to identify mechanistically relevant, non-DE regulators generalizes beyond IAV, we applied the method to a second infectious disease model: SARS-CoV-2 (Supplementary Fig. S17). Using an RNA-seq dataset of SARS-CoV-2–infected samples, we found that DENetwork again identified a large number of functionally relevant non-DE genes occupying central positions in the reconstructed signaling network. Reactome-based pathway enrichment showed that the DENetwork-derived genes were significantly enriched in “SARS-CoV-2 Infection” *$(\mathit {P} = 5.0 \times 10^{-21}),$* “Viral Infection” *$(\mathit {P} = 5.0 \times 10^{-26}),$* and other virus-specific pathways. In contrast, the DESeq2-derived genes were mainly enriched for general immune response pathways, such as “Interferon signaling” *$(\mathit {P} = 1.1 \times 10^{-6})$* and “Cytokine signaling” *$(\mathit {P} = 7.1 \times 10^{-4}),$* with limited evidence of SARS-CoV-2–specific biology. These findings further support the ability of DENetwork to uncover regulatory mechanisms in exogenous infectious diseases by identifying critical regulators that lack strong transcriptional changes.

These findings demonstrate that DENetwork identifies functionally important genes that are not DE, yet are essential for mediating signal flow from viral receptors to transcriptional responses. By reconstructing infection-specific signaling cascades, it reveals critical genes missed by expression-based methods and provides a mechanistic framework to dissect host responses in infectious diseases.

### DENetwork identifies critical immune genes in *Alox15^−^*^/−^ macrophages

**Figure 3.**
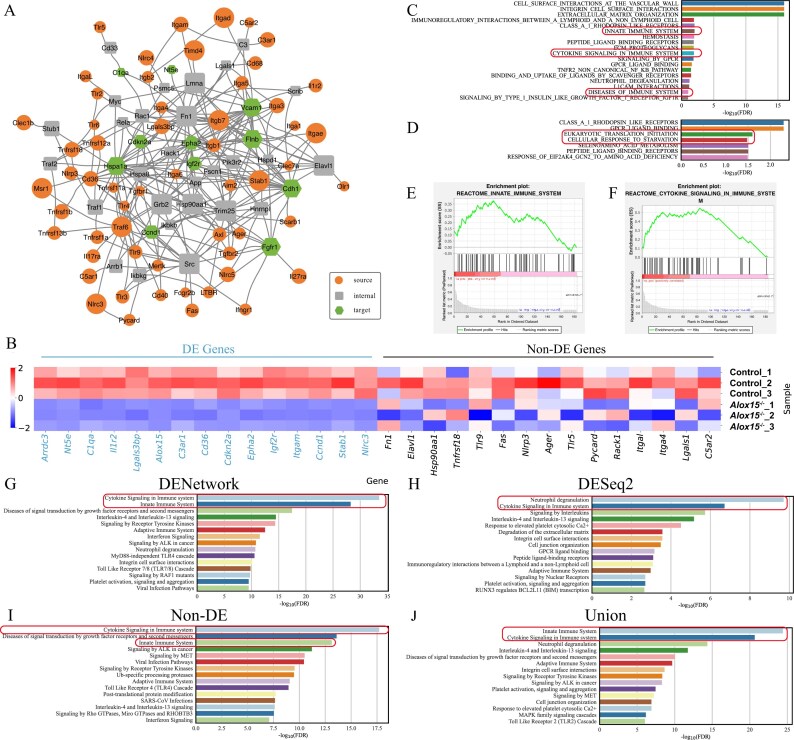
DENetwork identifies critical genes in macrophages of *Alox15^−^*^/−^ mice. (**A**) Network graph visualizing the top 100 genes within the final DENetwork-derived network. Nodes not contributing to network connections were excluded. Source nodes (receptors), internal nodes, and target nodes (upregulated DE genes) are represented by ellipses, rectangles, and hexagons, respectively. A larger node size signifies a higher impact (ranking) in the network. (**B**) A heatmap displaying the expression profiles of 15 upregulated DE genes and 15 non-DE genes in control and *Alox15^−^*^/−^ macrophage samples. Downregulated genes are highlighted in blue; non-DE genes are marked in black. (**C, D**) GSEAPreRanked analysis results. (C) uses the DENetwork-based ranking (descending node scores), while (D) uses the DESeq2-based ranking (ascending *P*). Representative pathways highly relevant to the *Alox15^−^*^/−^ macrophage dataset are highlighted with boxes. GSEA enrichment plots of representative pathways identified by DENetwork ranking (**E**) and DESeq2 ranking (**F**). Reactome pathway enrichment analysis performed on four distinct gene sets: all genes in the DENetwork-derived network (**G**), upregulated DE genes identified by DESeq2 (**H**), non-DE genes within this network (**I**), and the union of DENetwork and DESeq2 gene sets (**J**). Each panel displays the top 15 most significant pathways, with immune-related pathways highlighted by boxes.

To systematically investigate the molecular mechanisms underlying the dysregulated immune response in *Alox15^−^*^/− ^macrophages, we applied DENetwork to an RNA-seq dataset comprising three wild-type and three *Alox15^−^*^/− ^samples. One *Alox15^−^*^/−^ sample was excluded from the original set based on principal component analysis (Supplementary Fig. S18) due to suspected mislabeling. Receptor genes curated from CellTalkDB [17] were designated as source nodes, while significantly downregulated DE genes identified by DESeq2 were used as target nodes, as they directly reflect the loss of *Alox15* function and the resulting immune imbalance in the knockout context [42]. Based on this input, DENetwork reconstructed a gene regulatory network specific to *Alox15^−^*^/−^ macrophages. After statistical filtering, a total of 406 genes were retained as putative key nodes (all results were obtained using a consistent set of statistical thresholds and hyperparameter settings). Consistent with the approach used in the Influenza dataset, we extracted a representative subnetwork consisting of the top 100 genes ranked by network importance scores to visualize the reconstructed DENetwork network. Among these top 100 genes, nine were disconnected from the main component and were excluded. The resulting final visualized network (Fig. 3A) contained 91 connected nodes, including 44 source nodes (curated receptors, of which 34 were DE but still labeled as source nodes, and the remaining 10 were non-DE), 11 target nodes (downregulated DE genes), and 35 internal signaling molecules (all non-DE). These node categories were distinguished by shape and color, and node size reflected their relative ranking in the network, with larger nodes indicating higher inferred importance. The complete DENetwork-derived network, comprising all 406 prioritized genes, is provided in Supplementary Table S2 for comprehensive reference and reproducibility.

Among the top-ranked genes prioritized by DENetwork, there is a highly significant enrichment of downregulated DE genes identified by DESeq2 (hypergeometric enrichment $\mathit P = 2.37 \times 10^{-290}$; Fig. 3B, left), including *Arrdc3, Nt5e, C1qa, Cd36, Cdkn2a, Itgam*, and *Ccnd1*. These genes play critical roles in macrophage differentiation, phagocytic activity, and inflammation resolution, and exhibited consistent downregulation across all *Alox15^−^*^/−^ samples, consistent with DESeq2 results [43–48]. For example, *C1qa* is involved in apoptotic cell clearance and immune tolerance [45]; *Cd36* facilitates lipid uptake and scavenger function [46]; and *Nt5e* suppresses inflammation via adenosine signaling [44].

More notably, DENetwork also prioritized a set of non-DE genes that were not identified by DESeq2 but are functionally relevant in the context of macrophage biology (Fig. 3B, right). These include *Fn1, Elavl1, Hsp90aa1, Tnfrsf18, Tlr9, Fas, Nlrp3, Ager*, and *Rack1*. Although these genes did not exhibit statistically significant expression changes, they are well-documented players in immune signaling, inflammasome activation, and cytokine production. For instance, *Fn1* (Fibronectin 1) supports extracellular matrix (ECM) remodeling and immune cell adhesion [49]; *Elavl1* (HuR) stabilizes transcripts of key inflammatory mediators [50]; and *Nlrp3* and *Tlr9* are essential components of pathogen sensing and inflammasome assembly [51].

The identification of functionally important genes that are not significantly altered at the transcript level highlights the unique advantage of DENetwork. Under stress conditions such as dysregulated inflammation, some key genes may exert their effects through translational control, protein stability regulation, post-translational modifications, or amplification within signaling pathways, without transcriptional upregulation or downregulation. Because conventional methods like DESeq2 rely on transcript-level differences, these genes are often overlooked. DENetwork instead prioritizes genes that are critical for propagating signal flow from receptor source nodes to transcriptionally altered target nodes. This network-based approach enables the detection of mechanistically important genes that shape cellular responses under *Alox15^−^*^/−^, including impaired phagocytic capacity, reduced chemotaxis, and a shift from anti-inflammatory to pro-inflammatory macrophage states.

To confirm that the identified non-DE genes were not false negatives due to insufficient statistical power, we further validated them by relaxing the $\log _2(\mathrm{FC})$ threshold used in the differential expression analysis (Supplementary Fig. S14) and applying the non-parametric two-sided Wilcoxon rank-sum test (Supplementary Fig. S15). In both cases, the selected genes remained statistically non-significant, supporting the robustness of DENetwork’s prioritization.

To further assess the functional relevance of DENetwork-prioritized genes, we employed two complementary enrichment approaches: preranked GSEA, which examines the global biological coherence of the entire DENetwork-derived gene ranking, and hypergeometric enrichment testing, which evaluates the overrepresentation of biological pathways within the top-ranked gene subset identified by DENetwork.

GSEA using DENetwork-ranked genes (Fig. 3C) revealed strong enrichment for immune-related pathways, including Innate Immune System (*P* = 1.0 × 10^–2^), Cytokine Signaling in Immune system (*P* = 1.2 × 10^–2^), and Diseases of Immune System (*P* = 5.2 × 10^–2^), indicating that the network-derived ranking effectively captures key immune-related genes. In contrast, GSEA based on DESeq2 *P* rankings (Fig. 3D) predominantly enriched for stress response and metabolic processes, such as Cellular Response to Starvation (*P* = 3.3 × 10^–2^) and Eukaryotic Translation Initiation (*P* = 2.8 × 10^–2^) , with limited detection of immune signaling. This distinction was further illustrated in enrichment plots for representative pathways (Fig. 3E and F), where DENetwork showed leading-edge enrichment for immune-relevant processes.

Consistent with the GSEA results, Reactome enrichment analysis confirmed the functional relevance of DENetwork-prioritized genes (Fig. 3G–J). The full set of DENetwork-identified genes (Fig. 3G) showed strong enrichment for immune-related and signaling pathways, including Cytokine Signaling in Immune system ($\mathit {P} = 3.2 \times 10^{-34}$), Innate Immune System (*P* = 3.2 × 10^–29^), and Adaptive Immune System (*P* = 5.0 × 10^–13^). In contrast, the DESeq2-only gene set (Fig. 3H) was mainly enriched for downstream effector functions, such as Neutrophil degranulation (*P* = 2.5 × 10^–10^) and Signaling by Interleukins (*P* = 1.6 × 10^–6^), but failed to capture upstream signaling modules and receptor-mediated cascades.

Notably, even after removing all DE genes identified by DESeq2, the DENetwork-derived non-DE subset (Fig. 3I) retained strong enrichment for immune-related pathways, including Cytokine Signaling in Immune system (*P* = 1.0 × 10^–13^) and Viral Infection Pathways (*P* = 3.2 × 10^–11^).When DENetwork and DESeq2 gene sets were combined (union set = non-DE + DE genes, Fig. 3J), enrichment for immune pathways was partially recovered, though the signal was predominantly driven by non-DE genes identified by DENetwork. This union strategy, by integrating regulatory and transcriptional components, provided broader and more biologically coherent enrichment than either method alone, highlighting the complementary and synergistic value of combining DENetwork and DESeq2 results.

DENetwork expands the discovery space of immune-related pathways by identifying non-DE genes that are central to macrophage signaling cascades in *Alox15^−^*^/−^ mice. By integrating network topology with gene expression data, it uncovers functionally important genes missed by traditional DE analysis and reveals altered signaling circuits underlying macrophage dysfunction and inflammation.

### DENetwork identifies key genes in HuR-dependent pathways in lung fibroblasts

HuR, encoded by *ELAVL1*, is a member of the Hu/ELAV family of RNA-binding proteins. It regulates gene expression by binding to adenylate- and uridylate-rich elements in the 3′ untranslated regions of target messenger RNAs (mRNAs), thereby promoting mRNA stability [52]. In lung tissue, HuR is broadly expressed in epithelial cells and fibroblasts [53, 54], and plays an important role in promoting fibroblast differentiation and ECM production through TGF-–mediated signaling [55, 56]. To further investigate the functional role of HuR in lung fibroblasts, we used siRNA to silence *ELAVL1* in primary human lung fibroblasts, achieving >50% reduction in transcript levels [55]. RNA-seq was subsequently performed on both HuR knockdown and control samples to assess the global transcriptomic changes induced by HuR silencing. We then applied DENetwork to this dataset, which consisted of six samples. Receptor genes curated from CellTalkDB [17] were designated as source nodes, while significantly downregulated DE genes identified by DESeq2 were used as target nodes.

**Figure 4.**
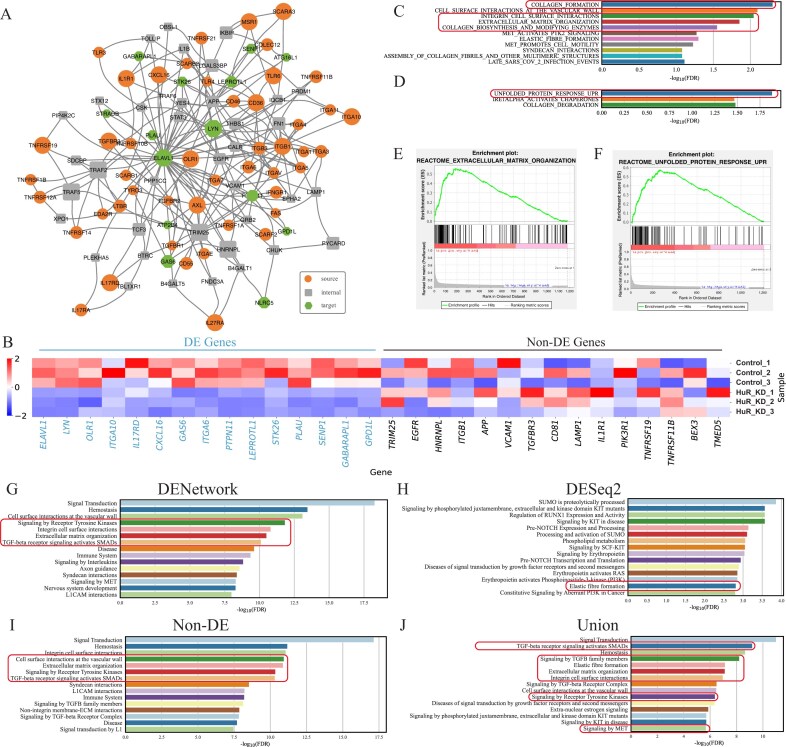
DENetwork identifies fibroblast-relevant genes and pathways controlled by HuR. (**A**) Network graph visualizing the top 100 genes within the final DENetwork-derived network. Nodes not contributing to network connections were excluded. Source nodes (receptors), internal nodes, and target nodes (downregulated DE genes) are represented by ellipses, rectangles, and hexagons, respectively. A larger node size signifies a higher impact (ranking) in the network. (**B**) Heatmap showing the expression profiles of 15 downregulated DE genes and 15 non-DE genes across control (WT) and HuR knockdown (KD_HuR) fibroblast samples. Downregulated genes are highlighted in blue; non-DE genes are marked in black. (**C, D**) GSEAPreRanked analysis results. (C) uses the DENetwork-based ranking (descending node scores), while (D) uses the DESeq2-based ranking (ascending *P*). Representative pathways highly relevant to fibroblast biology are highlighted with boxes. GSEA enrichment plots of representative pathways identified by DENetwork ranking (E) and DESeq2 ranking (F). Reactome pathway enrichment analysis performed on four distinct gene sets: all genes in the DENetwork-derived network (**G**), downregulated DE genes identified by DESeq2 (**H**), non-DE genes within this network (**I**), and the union of DENetwork and DESeq2 gene sets (**J**). Each panel displays the top 15 most significant pathways, with fibroblast-relevant pathways highlighted by boxes.

Based on this input, DENetwork reconstructed a gene interaction network specific to HuR knockdown. After statistical filtering, a total of 317 genes were retained as key nodes (all results were obtained using a consistent set of statistical thresholds and hyperparameter settings). Following the same approach as in the Influenza dataset, we extracted a representative subnetwork comprising the top 100 genes ranked by network importance scores to visualize the DENetwork-derived network. Among these top 100 genes, seven were disconnected from the main component and were excluded. As a result, the final visualized network (Fig. 4A) contained 93 connected nodes, including 47 source nodes (receptor genes, of which 40 were DE but still labeled as source nodes, and 7 were non-DE), 12 target nodes (downregulated DE genes), and 34 internal genes (all non-DE). These three categories of nodes were distinguished by shape and color, and node size reflected their network importance scores. The complete DENetwork-derived network, comprising all 317 prioritized genes, is provided in Supplementary Table S3.

Among the top-ranked genes prioritized by DENetwork, we observed a highly significant enrichment for DE genes identified by DESeq2 (hypergeometric enrichment $P < 1 \times 10^{-300}$), including *ELAVL1, LYN, OLR1, ITGA10, IL17RD, CXCL16, GAS6, ITGA6, PTPN11, LEPROTL1, STK26, PLAU, SENP1, GABARAPL1*, and *GPD1L* (Fig. 4B, left). Many of these genes are involved in ECM remodeling, integrin-mediated adhesion, and cytokine signaling, which are biological processes closely associated with fibroblast activation and fibrosis progression. For example, *ITGA6, GAS6*, and *PLAU* are known to participate in TGF-$\beta$–driven tissue remodeling [57–59], while *CXCL16* and *IL17RD* are implicated in immune cell signaling and inflammation [60, 61].

Importantly, DENetwork also uncovered a number of highly ranked genes that were not classified as DE by DESeq2 but are functionally relevant in fibroblast biology and fibrotic signaling (Fig. 4B, right). These non-DE but centrally ranked genes include *TRIM25, EGFR, HNRNPL, ITGB1, APP, VCAM1, TGFBR3, CD81, LAMP1, IL1R1, PIK3R1, TNFRSF19, TNFRSF11B, BEX3*, and *TMED5*. Although not differentially expressed, these genes are well-documented contributors to ECM remodeling, fibroblast–matrix interaction, and inflammatory signaling. For instance, *EGFR* and *TGFBR3* regulate TGF-beta–mediated fibrosis [62, 63]; *ITGB1* and *VCAM1* facilitate fibroblast adhesion to ECM [64, 65]; and *TRIM25*, an RNA-binding E3 ligase [66], influences post-transcriptional control and immune signaling.

These genes were not identified by conventional differential expression but emerged as critical nodes for mediating signal flow from receptors to DE targets. DENetwork does not rank genes by expression difference but by their necessity in transmitting signaling within the network. This design allows DENetwork to uncover functionally important genes that may exert their effects through post-transcriptional regulation, protein stability, or signaling cascades, which are mechanisms highly relevant to HuR function. These genes likely serve as key intermediaries mediating fibroblast remodeling responses to HuR knockdown.

To validate that these non-DE genes were not false negatives due to insufficient statistical power, we relaxed the $\log _2(\mathrm{FC})$ threshold in the DE analysis (Supplementary Fig. S14) and applied a non-parametric two-sided Wilcoxon rank-sum test (Supplementary Fig. S15). The selected genes remained statistically non-significant, confirming their classification and supporting the robustness of DENetwork.

To further assess the biological relevance of DENetwork-prioritized genes under HuR knockdown, we employed two complementary enrichment approaches: preranked GSEA, which examines the coherence of the full DENetwork gene ranking, and hypergeometric enrichment testing, which evaluates the overrepresentation of biological pathways within top-ranked gene subsets.

GSEA using DENetwork-ranked genes (Fig. 4C) revealed strong enrichment for pathways related to ECM remodeling and signal transduction, including Collagen formation (*P* = 5.0 × 10^–3^), Collagen biosynthesis and modifying enzymes (*P* = 2.8 × 10^–2^), Integrin cell surface interactions (*P* = 9 × 10^–3^), and ECM organization (*P* = 1.4 × 10^–2^). In contrast, GSEA using DESeq2 *P* rankings (Fig. 4D) highlighted general stress response pathways, such as the Unfolded Protein Response (*P* = 1.3 × 10^–2^), with minimal detection of ECM-specific processes. Enrichment plots (Fig. 4E and F) confirmed that ECM-related pathways were strongly concentrated at the top of the DENetwork ranking.

Reactome pathway enrichment further confirmed the functional relevance of DENetwork genes (Fig. 4G–J). The DENetwork-prioritized gene set (Fig. 4G) was enriched for core fibroblast signaling processes, including TGF-beta receptor signaling activates SMADs (*P* = 1.0 × 10^–10^), Signaling by Receptor Tyrosine Kinases (*P* = 5.0 × 10^–12^), and ECM organization (*P* = 4.0 × 10^–11^). Notably, even after removing all DESeq2-identified genes, the DENetwork non-DE subset (Fig. 4I) maintained enrichment for ECM- and TGF-–related pathways. In contrast, the DESeq2-only gene set (Fig. 4H) primarily enriched for processes unrelated to ECM, such as SUMOylation and KIT/NOTCH signaling. When both gene sets were combined (union set = non-DE + DE genes, Fig. 4J), enrichment for core fibroblast pathways was restored, driven primarily by the DENetwork-derived non-DE genes. This highlights the complementary contributions of regulatory (non-DE) and transcriptional (DE) components to the full biological signal.

DENetwork expands the discovery space of fibrosis-associated pathways by identifying non-DE genes that mediate signal flow from receptors to transcriptionally altered targets in HuR-knockdown fibroblasts. Through this network-based strategy, DENetwork recovers core fibrotic signaling modules missed by expression analysis alone, including TGF-$\beta$–related and ECM remodeling pathways. These findings suggest that HuR-dependent regulation of lung fibroblasts operates not only via transcriptional control, but also through post-transcriptional and signaling mechanisms, offering mechanistic insight and candidate targets for therapeutic intervention in fibrotic lung disease.

### DENetwork identifies key genes in receptor-mediated signaling pathways in liver cancer

Unlike infectious diseases, cancer is an endogenous disease whose initiation and progression depend on complex internal processes, including receptor-mediated signaling, intracellular regulatory networks, and microenvironmental remodeling. To assess DENetwork’s applicability in such contexts, we applied it to a hepatocellular carcinoma (HCC) RNA-seq dataset to investigate receptor-driven signaling networks. In addition, this dataset includes survival data from TCGA, enabling quantitative evaluation of gene prioritization strategies through survival analysis.

Specifically, we analyzed 14 samples from the GDC liver cancer cohort, comprising tumor tissues and matched adjacent non-tumor controls. Receptor genes curated from CellTalkDB [17] were designated as source nodes (Supplementary Fig. S13 shows significant association with a curated liver cancer gene set, $P = 9.5 \times 10^{-17}$), and upregulated DE genes identified by DESeq2 were used as target nodes.

**Figure 5.**
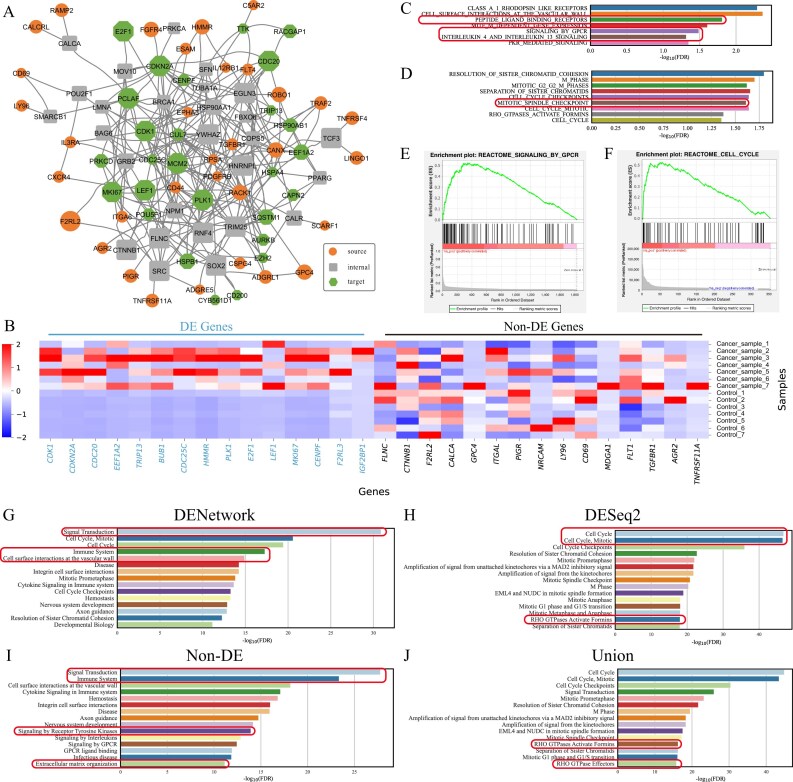
DENetwork identifies crucial DE and non-DE genes associated with liver cancer signaling. (**A**) Network graph visualizing the top 100 genes in the final DENetwork-derived network for the liver cancer dataset. Source nodes (receptors), internal nodes, and target nodes (upregulated DE genes) are represented by ellipses, rectangles, and hexagons, respectively. Nodes not contributing to network connections were excluded. A larger node size signifies a higher impact (ranking) in the network. (**B**) Heatmap showing the expression profiles of 15 upregulated DE genes and 15 non-DE genes across tumor and non-tumor samples. Upregulated genes are highlighted in blue; non-DE genes are marked in black. (**C, D**) GSEAPreRanked analysis results. (C) uses the DENetwork-based ranking (descending node scores), while (D) uses the DESeq2-based ranking (ascending *P*). Representative pathways highly relevant to liver cancer biology are highlighted with boxes. GSEA enrichment plots of representative pathways identified by DENetwork ranking (**E**) and DESeq2 ranking (**F**). Reactome pathway enrichment analysis performed on four distinct gene sets: all genes in the DENetwork-derived network (**G**), upregulated DE genes identified by DESeq2 (**H**), non-DE genes within this network (**I**), and the union of DENetwork and DESeq2 gene sets (**J**). Each panel displays the top 15 most significant pathways, with liver cancer–relevant pathways highlighted by boxes.

Based on this input, DENetwork reconstructed a liver cancer-associated signaling network comprising 467 genes that passed statistical filtering. As in previous analyses, we extracted a representative subnetwork of the top 100 genes ranked by network importance. After excluding 13 disconnected genes, the visualized network (Fig. 5A) contained 87 connected nodes: 30 source nodes (26 DE and 4 non-DE), 33 DE target genes, and 24 internal non-DE nodes. Node categories were distinguished by shape and color, and node size reflected importance scores. The full network is provided in Supplementary Table S4.

Among the top-ranked genes prioritized by DENetwork, we observed a highly significant enrichment of DE genes identified by DESeq2 (hypergeometric enrichment $P < 1 \times 10^{-300}$), including *CDK1, CDKN2A, CDC20, EEF1A2, TRIP13, BUB1, CDC25C, HMMR, PLK1, E2F1, LEF1, MKI67, CENPF, F2RL3*, and *IGF2BP1* (Fig. 5B, left). These genes are heavily involved in cell cycle regulation, mitotic progression, and tumor proliferation, which are hallmarks of HCC biology [67–72].

More importantly, DENetwork prioritized several top-ranked genes not classified as DE by DESeq2 but with known relevance to HCC (Fig. 5B, right). These include *FLNC, CTNNB1, F2RL2, CALCA, GPC4, ITGAL, PIGR, NRCAM, LY96, CD69, MDGA1, FLT1, TGFBR1, AGR2*, and *TNFRSF11A*. Many of these are implicated in oncogenic signaling (e.g. *CTNNB1* in WNT signaling [73]), EMT and fibrosis (*TGFBR1* [74]), angiogenesis and inflammation (*FLT1, LY96* [75, 76]), and immune infiltration (*ITGAL, FLNC* [77, 78]).

To ensure these genes were not missed due to limited power in expression analysis, we relaxed the $\log _2(\mathrm{FC})$ threshold (Supplementary Fig. S14) and applied the two-sided Wilcoxon rank-sum test (Supplementary Fig. S15). The genes remained statistically non-significant, supporting that DENetwork captures functionally important but non-DE genes.

To evaluate pathway-level relevance, we performed GSEA using the DENetwork ranking (Fig. 5C). It revealed enrichment for pathways involved in receptor signaling and tumor microenvironment remodeling, such as Signaling by GPCR ($P = 3.3 \times 10^{-2}$), Peptide ligand-binding receptors ($P = 1.7 \times 10^{-2}$), and Interleukin signaling ($P = 4.9 \times 10^{-2}$). In contrast, GSEA using DESeq2 (Fig. 5D) retrieved cell cycle–related pathways such as Mitotic spindle checkpoint ($P = 2.5 \times 10^{-2}$) and Sister chromatid cohesion ($P = 1.6 \times 10^{-2}$). Enrichment plots (Fig. 5E and F) illustrate this divergence, with DENetwork capturing extracellular signaling, while DESeq2 emphasizes proliferative changes.

Reactome pathway enrichment further validated these trends (Fig. 5G–J). The DENetwork gene set (Fig. 5G) was enriched for both signal transduction and immune signaling. The DESeq2-only set (Fig. 5H) was dominated by cell cycle and Rho GTPase-related cytoskeletal pathways, whereas the DENetwork non-DE subset (Fig. 5I) still captured receptor tyrosine kinase signaling, ECM organization, and immune system pathways. The union set (union set = non-DE + DE genes, Fig. 5J) restored broader pathway coverage. However, key microenvironmental and signaling pathways remained largely driven by DENetwork-derived genes, highlighting the complementary contributions of non-DE regulators and DE targets. These results demonstrate that DENetwork complements traditional DE analysis by identifying non-DE but biologically important genes involved in receptor signaling and microenvironmental interactions. This network-based strategy offers deeper insight into tumor signaling architecture, providing a valuable framework for exploring HCC pathogenesis and prioritizing potential therapeutic targets.

### DENetwork outperforms generic DE and network-based methods in benchmarking tests

**Figure 6.**
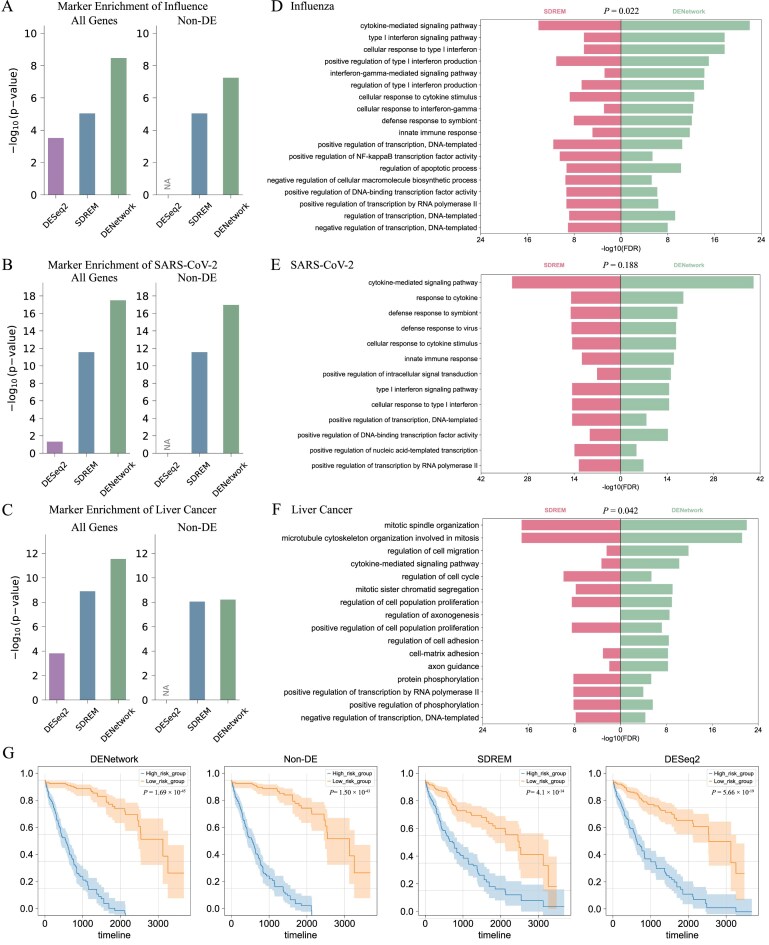
DENetwork outperforms generic DE and network-based methods in benchmarking tests. Bar plots showing the hypergeometric enrichment of known marker genes for Influenza (**A**), SARS-CoV-2 (**B**), and liver cancer (**C**) across gene sets identified by DESeq2, SDREM, DENetwork, and their respective non-DE subsets. Comparison of GOBP enrichment significance between DENetwork+DESeq2 and SDREM+DESeq2 for Influenza (**D**), SARS-CoV-2 (**E**), and liver cancer (**F**). Significance was assessed on the $-\log _{10}(\mathrm{FDR})$  *P* of the union of the top 10 pathways from both methods; the resulting *P* in each panel reflects this overall paired comparison. All tests were performed using a one-sided Wilcoxon signed-rank test. (**G**) Kaplan–Meier survival curves comparing high-risk and low-risk patient groups stratified by gene sets identified by DENetwork, its non-DE subset, SDREM, and DESeq2 in the liver cancer cohort.

To systematically evaluate the performance of DENetwork against other network-based gene prioritization methods, we designed a three-pronged benchmarking analysis. First, we assessed how well each method recapitulated known disease-associated genes. Specifically, we compared the overlap of prioritized gene sets identified by DENetwork, the non-DE subset (genes identified by DENetwork but not by DESeq2), SDREM, and DESeq2 with curated disease marker gene sets for Influenza, SARS-CoV-2, and liver cancer obtained from public databases. We applied hypergeometric enrichment analysis to quantify the significance of these overlaps. As shown in Fig. 6A–C, DENetwork consistently exhibited the strongest enrichment across all three diseases. Critically, even after removing all DE genes, the non-DE subset identified by DENetwork retained substantial enrichment for known markers, particularly in the Influenza dataset (Fig. 6A), where 18 out of the top 100 DENetwork-prioritized genes were non-DE yet significantly enriched for antiviral relevance. In contrast, neither SDREM’s non-DE subset nor DESeq2 (which cannot identify non-DE genes) recovered such hidden yet functionally essential components. This uniquely positions DENetwork to uncover regulatory drivers that are missed by methods reliant on expression thresholds alone. To further illustrate the relationships among methods, we also generated Venn diagrams (Supplementary Fig. S19) showing the overlaps of prioritized genes across DENetwork, SDREM, and DESeq2 for each dataset. From these diagrams, we observed that DENetwork and SDREM share a large overlap, whereas both methods have much less overlap with DESeq2, highlighting the distinct gene sets identified by differential expression analysis compared to network-based approaches.

We next examined whether the prioritized gene sets exhibit coherent biological function by evaluating GOBP enrichment. To compare biological interpretability, we analyzed the union of each method’s prioritized genes with DESeq2 and assessed the significance of the top enriched pathways. As shown in Fig. 6D–F, DENetwork consistently produced more specific and mechanistically relevant terms across all datasets. In the Influenza dataset, DENetwork recovered well-established antiviral responses such as type I interferon signaling pathway, defense response to virus, and cellular response to type I interferon, while SDREM’s results were confined to broader cytokine signaling categories. Similar patterns were observed in the SARS-CoV-2 dataset, where DENetwork identified canonical viral response programs with stronger significance. For the liver cancer dataset, DENetwork retrieved key oncogenic and microenvironmental processes, including positive regulation of cell migration, signal transduction, and regulation of cell proliferation, whereas SDREM largely returned generic cell cycle-related pathways. To further assess whether these observed differences in pathway enrichment were statistically significant, we quantitatively compared enrichment results across methods by evaluating the distributions of $-\log _{10}(\mathrm{FDR})$ values using a one-sided Wilcoxon signed-rank test, with the reported $P$ reflecting this directional comparison. These results underscore that DENetwork captures more mechanistically coherent and disease-relevant biological processes.

To determine whether DENetwork’s prioritization translates to clinical relevance, we evaluated the prognostic performance of each gene set using survival data from a liver cancer cohort. Patients were stratified into high- and low-risk groups based on expression profiles derived from the genes selected by each method. As illustrated in Fig. 6G, DENetwork achieved the most significant survival stratification (log-rank $P = 1.69 \times 10^{-45}$), far outperforming DESeq2 ($P = 5.66 \times 10^{-19}$), and SDREM ($P = 4.1 \times 10^{-14}$). Notably, the non-DE subset alone from DENetwork exhibited strong prognostic value ($P = 1.50 \times 10^{-38}$), further validating the functional and clinical importance of transcriptionally silent regulators prioritized by our method.

Taken together, these benchmarking results demonstrate that DENetwork outperforms both traditional differential expression tools and generic network-based methods in identifying biologically and clinically important genes. By prioritizing genes based on their topological role in signaling, rather than solely on differential expression, DENetwork captures a critical layer of mechanistic insight that enhances both discovery and translational applications.

## Discussion

In this study, we developed DENetwork, a graphical model designed to improve the interpretation of RNA-seq data by uncovering key regulatory genes underlying condition-specific molecular changes, thereby expanding its applicability across a wide range of biological contexts. By integrating gene expression profiles with prior knowledge of receptor–ligand interactions, intracellular signaling cascades (we selected HIPPIE as the default PPI database due to its emphasis on experimentally validated interactions and its superior performance in our empirical evaluations), and transcriptional regulation, DENetwork constructs condition-specific signaling networks and ranks genes based on their contribution to network-wide signal propagation.

The contributions of DENetwork are fourfold. First, it goes beyond association-based approaches, such as differential expression analysis (e.g., DESeq2), which detect statistical correlations between gene expression and condition but cannot distinguish upstream drivers from downstream consequences. DENetwork instead employs a network-based *in silico* perturbation framework, where each gene is virtually knocked out and its impact on global signal flow is quantified. This allows us to approximate causal influence by measuring how much each gene contributes to driving condition-specific transcriptomic differences. Second, this perturbation-based scoring enables the identification of both DE and non-DE genes that play functionally important roles in shaping cellular state transitions, highlighting likely upstream regulators such as receptors, signaling proteins, and TFs. Third, DENetwork does not merely produce a list of ranked genes and scores; it also reconstructs the upstream signaling network that mechanistically links candidate regulators to observed gene expression differences. This level of functional interpretation is absent in conventional differential expression analyses, which lack regulatory context. Fourth, although prior methods such as SDREM also aim to recover upstream signaling regulators, SDREM is specifically designed for time-series transcriptomic data and relies on temporal expression trajectories to model signaling dynamics using dynamic Bayesian networks or Hidden Markov Models. This temporal modeling enables SDREM to infer regulator-target relationships over time but limits its applicability to static RNA-seq data, where such time-resolved information is unavailable. In contrast, DENetwork is purpose-built for static, cross-sectional transcriptomic comparisons, which are common in most biological and clinical studies, and infers condition-specific signaling networks directly from snapshot expression profiles. This design choice makes DENetwork more broadly applicable to real-world datasets where time series are not available or practical to collect.

These contributions are underscored by the identification of *Elavl1* (HuR) as a dominant, non-DE gene in macrophages from *Alox15*^-/-^ mice. *Alox15* encodes 15-lipoxygenase, an enzyme that generates the pro-resolving eicosanoid lipoxin A4 and operates in close coordination with cyclooxygenases (COX), which produce prostaglandins and thromboxanes. HuR has been identified as a key regulator of eicosanoid production via stabilization of *Ptgs2*, the gene encoding COX-2 [79, 80]. It is abundantly expressed in macrophages across various tissues [81, 82], where it plays a central role in macrophage polarization and the suppression of inflammation [83]. The identification of *Elavl1* by DENetwork, despite its non-differential expression, demonstrates the method’s ability to detect upstream, functionally relevant regulators that may be missed by conventional approaches. This biological insight was further supported by experimental validation in lung fibroblasts, where HuR was found to influence signaling pathways involving TGF-$\beta$ and receptor tyrosine kinases (RTKs), key regulators of fibroblast activation, ECM remodeling, and wound healing. These findings align with previous studies, including our own, showing that HuR promotes the expression of collagen and matrix genes in response to TGF-$\beta$ [84–86]. Notably, several HuR-regulated genes were activated even in the absence of exogenous TGF-$\beta$, underscoring HuR’s intrinsic role in fibroblast biology. Together, these results serve as biological evidence supporting the functionality and effectiveness of DENetwork in uncovering context-relevant, mechanistically important regulators beyond what differential expression alone can reveal.

While DENetwork provides a powerful framework for identifying functionally important regulators, several limitations remain. Its reliance on curated biological interaction networks may result in incomplete coverage or in signaling interactions that are not fully representative of the specific cell type, tissue, or condition being studied. Future extensions could incorporate dynamic or tissue-specific network inference and integrate additional omics layers such as proteomics or epigenomics to improve accuracy and biological relevance. Moreover, although DENetwork approximates gene influence through *in silico* perturbation, this approach provides only an implicitly causal estimate of signaling importance. Combining this strategy with formal causal inference frameworks or experimental perturbation data could further enhance the mechanistic interpretability of the results and support translational applications.

By expanding the analytical scope beyond differential expression and adopting an influence-based, network-aware perspective, DENetwork introduces a shift in how transcriptomic data can be analyzed and interpreted. This framework enables researchers to identify upstream, mechanistically relevant regulators, regardless of their differential expression status, that shape cellular functions. DENetwork may prove valuable for systems biologists seeking to uncover regulatory hierarchies, for translational researchers prioritizing therapeutic targets, and for data analysts aiming to derive more mechanistic insight from static RNA-seq datasets. As such, DENetwork has the potential to advance systems-level understanding and target discovery across a wide range of biological contexts.

## Supplementary Material

gkaf1350_Supplemental_Files

## Data Availability

The DENetwork software, together with a comprehensive user manual and illustrative examples, is freely accessible on GitHub at https://github.com/mcgilldinglab/DENetwork and Zenodo at https://doi.org/10.5281/zenodo.17211767 [87]. We evaluated DENetwork using five publicly available bulk RNA-seq datasets. The Influenza A virus (IAV) infection dataset is available in the Gene Expression Omnibus (GEO) under accession number GSE192528. The SARS-CoV-2 infection dataset in human lung tissue can be accessed via GEO under accession number GSE147507. The macrophage Alox15 knockout (Alox15^–/–^) dataset is available in GEO under accession number GSE216531. The liver cancer (hepatocellular carcinoma) dataset was obtained from The Cancer Genome Atlas (TCGA) through the Genomic Data Commons (GDC) portal (https://portal.gdc.cancer.gov/). In addition, the HuR knockdown (lung fibroblast) dataset has been deposited in Zenodo at https://doi.org/10.5281/zenodo.17211957. All processed RNA-seq datasets associated with this study are deposited on Zenodo at https://doi.org/10.5281/zenodo.17211695 [88].

## References

[1] Bass A, Liu Y, Dakshanamurthy S. Single-cell and bulk RNA-seq profiling of COVID-19 patients reveal immune and inflammatory mechanisms of infection-induced organ damage. Viruses; 2021; 13: 2418. 10.3390/v13122418. 34960687 PMC8706409

[2] Byron SA, Keuren-Jensen KRV, Engelthaler DM et al. Translating RNA sequencing into clinical diagnostics: opportunities and challenges. Nat Rev Genet; 2016; 17: 257–71. 10.1038/nrg2484. 26996076 PMC7097555

[3] Wang Z, Gerstein M, Snyder M. RNA-seq: a revolutionary tool for transcriptomics. Nat Rev Genet; 2009; 10: 57–63. 10.1038/nrg2484. 19015660 PMC2949280

[4] Love MI, Huber W, Anders S. Moderated estimation of fold change and dispersion for RNA-seq data with DESeq2. Genome Biol; 2014; 15:550. 10.1186/s13059-014-0550-8. 25516281 PMC4302049

[5] Chen Y, Lun ATL, Smyth GK. From reads to genes to pathways: differential expression analysis of RNA-seq experiments using Rsubread and the edgeR quasi-likelihood pipeline. F1000Research; 2016; 5: 1438. 10.12688/f1000research.8987.2. 27508061 PMC4934518

[6] Hoque MN, Sarkar MMH, Khan MA et al. Differential gene expression profiling reveals potential biomarkers and pharmacological compounds against SARS-CoV-2: insights from machine learning and bioinformatics approaches. Front Immunol. 2022;13:918692. 10.3389/fimmu.2022.918692. 36059456 PMC9429819

[7] Babu G, Nobel FA. Identification of differentially expressed genes and their major pathways among the patient with COVID-19, cystic fibrosis, and chronic kidney disease. Inform Med Unlocked. 2022;32:101038. 10.1016/j.imu.2022.101038. 35966126 PMC9357445

[8] Li QS, Muynck LD. Differentially expressed genes in Alzheimer’s disease highlighting the roles of microglia genes including OLR1 and astrocyte gene CDK2AP1. Brain Behav Immun Health. 2021;13:100227. 10.1016/j.bbih.2021.100227. 34589742 PMC8474442

[9] min Xue J, Liu Y, hong Wan L et al. Comprehensive analysis of differential gene expression to identify common gene signatures in multiple cancers. Med Sci Monit. 2020;26:e919953. 10.12659/MSM.919953. 32035007 PMC7027371

[10] Zhang N, Bao YJ, Tong AHY et al. Whole transcriptome analysis reveals differential gene expression profile reflecting macrophage polarization in response to influenza A H5N1 virus infection. BMC Med Genomics. 2018;11:20. 10.1186/s12920-018-0335-0. 29475453 PMC6389164

[11] Merikangas AK, Shelly M, Knighton A et al. What genes are differentially expressed in individuals with schizophrenia? A systematic review. Mol Psychiatr. 2022;27:1373–83. 10.1038/s41380-021-01420-7. PMC9095490 35091668

[12] Blanco-Melo D, Nilsson-Payant BE, Liu WC et al. Imbalanced host response to SARS-CoV-2 drives development of COVID-19. Cell. 2020;181:1036–45. 10.1016/j.cell.2020.04.026. 32416070 PMC7227586

[13] Gómez I, López MC, Egui A et al. Differential expression profile of genes involved in the immune response associated to progression of chronic Chagas disease. PLoS Negl Trop Dis. 2023;17:e0011474. 10.1371/journal.pntd.0011474. 37440604 PMC10368263

[14] Zheng Y, Sun C, Zhang X et al. Missense mutations in CRX homeodomain cause dominant retinopathies through two distinct mechanisms. Elife. 2023;12:RP87147. 10.7554/eLife.87147. 37963072 PMC10645426

[15] Jain S, Gitter A, Bar-Joseph Z. Multitask learning of signaling and regulatory networks with application to studying human response to flu. PLoS Comput Biol. 2014;10:e1003943. 10.1371/journal.pcbi.1003943. 25522349 PMC4270428

[16] Ding J, Hostallero DE, El Khili MR et al. A network-informed analysis of SARS-CoV-2 and hemophagocytic lymphohistiocytosis genes’ interactions points to Neutrophil extracellular traps as mediators of thrombosis in COVID-19. PLoS Comput Biol. 2021;17:e1008810. 10.1371/journal.pcbi.1008810. 33684134 PMC7971900

[17] Shao X, Liao J, Li C et al. CellTalkDB: a manually curated database of ligand–receptor interactions in humans and mice. Brief Bioinformatics. 2021;22:bbaa269 10.1093/bib/bbaa269. 33147626

[18] Alanis-Lobato G, Andrade-Navarro MA, Schaefer MH. HIPPIE v2.0: enhancing meaningfulness and reliability of protein–protein interaction networks. Nucleic Acids Res. 2016;45:gkw985. 10.1093/nar/gkw985. PMC5210659 27794551

[19] Franceschini A, Szklarczyk D, Frankild S et al. STRING v9. 1: protein–protein interaction networks, with increased coverage and integration. Nucleic Acids Res. 2012;41:D808–15. 10.1093/nar/gks1094. 23203871 PMC3531103

[20] Ding J, Hagood JS, Ambalavanan N et al. iDREM: interactive visualization of dynamic regulatory networks. PLOS Comput Biol. 2018;14:e1006019. 10.1371/journal.pcbi.1006019. 29538379 PMC5868853

[21] Hagberg A, Swart PJ, Schult DA. Exploring network structure, dynamics, and function using NetworkX. 2008. 10.25080/TCWV9851.

[22] Gitter A, Bar-Joseph Z. Identifying proteins controlling key disease signaling pathways. Bioinformatics. 2013:29:i227–i236. 10.1093/bioinformatics/btt241. 23812988 PMC3694658

[23] Subramanian A, Tamayo P, Mootha VK et al. Gene set enrichment analysis: a knowledge-based approach for interpreting genome-wide expression profiles. Proc Natl Acad Sci. 2005;102:15545–50. 10.1073/pnas.0506580102. 16199517 PMC1239896

[24] Aloufi N, Traboulsi H, Ding J et al. Angiotensin-converting enzyme 2 expression in COPD and IPF fibroblasts: the forgotten cell in COVID-19. Am J Physiol Lung Cell Mol Physiol. 2021;320:L152–7. 10.1152/ajplung.00455.2020. 33112187 PMC7869954

[25] Andrews S . FastQC: A Quality Control tool for High Throughput Sequence Data. http://www.bioinformatics.babraham.ac.uk/projects/fastqc/.

[26] Ewels P, Magnusson M, Lundin S et al. MultiQC: summarize analysis results for multiple tools and samples in a single report. Bioinformatics. 2016;32:3047–8. 10.1093/bioinformatics/btw354. 27312411 PMC5039924

[27] Dobin A, Davis CA, Schlesinger F et al. STAR: ultrafast universal RNA-seq aligner. Bioinformatics. 2013;29:15–21. 10.1093/bioinformatics/bts635. 23104886 PMC3530905

[28] Li H, Handsaker B, Wysoker A et al. The Sequence Alignment/Map format and SAMtools. Bioinformatics. 2009;25:2078–9. 10.1093/bioinformatics/btp352. 19505943 PMC2723002

[29] Broad Institute . Picard toolkit. http://broadinstitute.github.io/picard/.

[30] Liao Y, Smyth GK, Shi W. featureCounts: an efficient general purpose program for assigning sequence reads to genomic features. Bioinformatics. 2014;30:923–30. 10.1093/bioinformatics/btt656. 24227677

[31] Love MI, Huber W, Anders S. Moderated estimation of fold change and dispersion for RNA-seq data with DESeq2. Genome Biol. 2014;15:1–21. 10.1186/s13059-014-0550-8. PMC4302049 25516281

[32] Langfelder P, Horvath S. WGCNA: an R package for weighted correlation network analysis. BMC Bioinformatics. 2008;9:1–13. 10.1186/1471-2105-9-559. 19114008 PMC2631488

[33] Betakova T, Kostrabova A, Lachova V et al. Cytokines induced during influenza virus infection. Curr Pharm Des. 2017;23:2616–22. 10.2174/1381612823666170316123736. 28302021

[34] Antunes I, Kassiotis G. Suppression of innate immune pathology by regulatory T cells during Influenza A virus infection of immunodeficient mice. J Virol. 2010;84:12564–75. 10.1128/JVI.01559-10. 20943986 PMC3004299

[35] Zhang S, Huo C, Xiao J et al. p-STAT1 regulates the influenza A virus replication and inflammatory response *in vitro* and *vivo*. Virology. 2019;537:110–20. 10.1016/j.virol.2019.08.023. 31493649

[36] Chen S, Zhang W, Wu Z et al. Goose Mx and OASL play vital roles in the antiviral effects of type I, II, and III interferon against newly emerging avian flavivirus. Front Immunol. 2017;8:1006. 10.3389/fimmu.2017.01006. 28878774 PMC5572330

[37] Hou J, Han L, Zhao Z et al. USP18 positively regulates innate antiviral immunity by promoting K63-linked polyubiquitination of MAVS. Nat Commun. 2021;12:2970. 10.1038/s41467-021-23219-4. 34016972 PMC8137702

[38] Tang L, Liu X, Wang C et al. USP18 promotes innate immune responses and apoptosis in influenza A virus-infected A549 cells via cGAS-STING pathway. Virology. 2023;585:240–7. 10.1016/j.virol.2023.06.012. 37422930

[39] Lenschow DJ, Lai C, Frias-Staheli N et al. IFN-stimulated gene 15 functions as a critical antiviral molecule against influenza, herpes, and Sindbis viruses. Proc Natl Acad Sci. 2007;104:1371–6. 10.1073/pnas.0607038104. 17227866 PMC1783119

[40] Dawson TC, Beck MA, Kuziel WA et al. Contrasting effects of CCR5 and CCR2 deficiency in the pulmonary inflammatory response to influenza A virus. Am J Pathol. 2000;156:1951–9. 10.1016/S0002-9440(10)65068-7. 10854218 PMC1850091

[41] Wahl A, Schafer F, Bardet W et al. HLA class I molecules reflect an altered host proteome after influenza virus infection. Hum Immunol. 2010;71:14–22. 10.1016/j.humimm.2009.08.012. 19748539 PMC2795087

[42] Pernet E, Sun S, Sarden N et al. Neonatal imprinting of alveolar macrophages via neutrophil-derived 12-HETE. Nature. 2023;614:530–8. 10.1038/s41586-022-05660-7. 36599368 PMC9945843

[43] Luo J, Tao H, Chen L et al. LncRNA MEG3 suppresses prostate cancer progression by mediating macrophage polarization via the miR-148a-3p/ARRDC3 signaling axis. Carcinogenesis. 2025;46:bgaf009. 10.1093/carcin/bgaf009. 40036590

[44] Minor M, Alcedo KP, Battaglia RA et al. Cell type-and tissue-specific functions of ecto-5′-nucleotidase (CD73). Am J Physiol Cell Physiol. 2019;317:C1079–92. 10.1152/ajpcell.00285.2019. 31461341 PMC6957383

[45] Horowitz A, Yu H, Pandey S et al. C1QA is an invariant biomarker for tissue macrophages. bioRxiv, 10.1101/2024.01.26.577475, 28 January 2024, preprint: not peer reviewed.

[46] Sanjurjo L, Amézaga N, Aran G et al. The human CD5L/AIM-CD36 axis: a novel autophagy inducer in macrophages that modulates inflammatory responses. Autophagy. 2015;11:487–502. 10.1080/15548627.2015.1017183. 25713983 PMC4502645

[47] Januszyk M . An Integrated Framework to Identify and Isolate Novel, Functionally Distinct Tissue Subpopulations Using High-Resolution Single Cell Analysis. Stanford University, 2015. https://purl.stanford.edu/rg047xt4187.

[48] Liang T, Chen J, Xu G et al. TYROBP, TLR4 and ITGAM regulated macrophages polarization and immune checkpoints expression in osteosarcoma. Sci Rep. 2021;11:19315. 10.1038/s41598-021-98637-x. 34588497 PMC8481262

[49] Dou F, Liu Q, Lv S et al. FN1 and TGFBI are key biomarkers of macrophage immune injury in diabetic kidney disease. Medicine. 2023;102:e35794. 10.1097/MD.0000000000035794. 37960829 PMC10637504

[50] Lu YC, Chang SH, Hafner M et al. ELAVL1 modulates transcriptome-wide miRNA binding in murine macrophages. Cell Rep. 2014;9:2330–43. 10.1016/j.celrep.2014.11.030. 25533351 PMC4277505

[51] Hoque R, Sohail M, Malik A et al. TLR9 and the NLRP3 inflammasome link acinar cell death with inflammation in acute pancreatitis. Gastroenterology. 2011;141:358–69. 10.1053/j.gastro.2011.03.041. 21439959 PMC3129497

[52] Myer VE . Identification of HuR as a protein implicated in AUUUA-mediated mRNA decay. EMBO J. 1997;16:2130–39. 10.1093/emboj/16.8.2130. 9155038 PMC1169815

[53] Zago M, Sheridan JA, Nair P et al. Aryl hydrocarbon receptor-dependent retention of nuclear HuR suppresses cigarette smoke-induced cyclooxygenase-2 expression independent of DNA-binding. PLoS One. 2013;8:e74953. 10.1371/journal.pone.0074953. 24086407 PMC3785509

[54] Fan J, Ishmael FT, Fang X et al. Chemokine transcripts as targets of the RNA-binding protein HuR in human airway epithelium. J Immunol. 2011;186:2482–94. 10.4049/jimmunol.0903634. 21220697 PMC3872785

[55] Al-Habeeb F, Aloufi N, Traboulsi H et al. Human antigen R promotes lung fibroblast differentiation to myofibroblasts and increases extracellular matrix production. J Cell Physiol. 2021;236:6836–51. 10.1002/jcp.30380. 33855709 PMC8390431

[56] Trivlidis J, Aloufi N, Al-Habeeb F et al. HuR drives lung fibroblast differentiation but not metabolic reprogramming in response to TGF-β and hypoxia. Resp Res. 2021;22:323. 10.1186/s12931-021-01916-4. PMC8715577 34963461

[57] Khademi R, Malekzadeh H, Bahrami S et al. Regulation and functions of α6-Integrin (CD49f) in cancer biology. Cancers. 2023;15:3466. 10.3390/cancers15133466. 37444576 PMC10341356

[58] Bellan M, Cittone MG, Tonello S et al. Gas6/TAM system: a key modulator of the interplay between inflammation and fibrosis. Int J Mol Sci. 2019;20:5070. 10.3390/ijms20205070. 31614787 PMC6834320

[59] Corley SM, Mendoza-Reinoso V, Giles N et al. Plau and Tgfbr3 are YAP-regulated genes that promote keratinocyte proliferation. Cell Death Dis. 2018;9:1106. 10.1038/s41419-018-1141-5. 30382077 PMC6208416

[60] Zhou C, Gao Y, Ding P et al. The role of CXCL family members in different diseases. Cell Death Disc. 2023;9:212. 10.1038/s41420-023-01524-9. PMC10314943 37393391

[61] Girondel C, Meloche S. Interleukin-17 receptor D in physiology, inflammation and cancer. Front Oncol. 2021;11:656004. 10.3389/fonc.2021.656004. 33833999 PMC8021910

[62] Zhuang S, Liu N. EGFR signaling in renal fibrosis. Kidney Int Suppl. 2014;4:70–4. 10.1038/kisup.2014.13. PMC4536964 26312153

[63] Pohlers D, Brenmoehl J, Löffler I et al. TGF-β and fibrosis in different organs—molecular pathway imprints. Biochim Biophys Acta. 2009;1792:746–56. 10.1016/j.bbadis.2009.06.004. 19539753

[64] Ji Q, Zhou L, Sui H et al. Primary tumors release ITGBL1-rich extracellular vesicles to promote distal metastatic tumor growth through fibroblast-niche formation. Nat Commun. 2020;11:1211. 10.1038/s41467-020-14869-x. 32139701 PMC7058049

[65] Zhou Z, Zhou Q, Wu X et al. VCAM-1 secreted from cancer-associated fibroblasts enhances the growth and invasion of lung cancer cells through AKT and MAPK signaling. Cancer Lett. 2020;473:62–73. 10.1016/j.canlet.2019.12.039. 31904479

[66] Choudhury NR, Heikel G, Trubitsyna M et al. RNA-binding activity of TRIM25 is mediated by its PRY/SPRY domain and is required for ubiquitination. BMC Biol. 2017;15:1–20. 10.1186/s12915-017-0444-9. 29117863 PMC5678581

[67] Hao L, Li S, Peng Q et al. Anti-malarial drug dihydroartemisinin downregulates the expression levels of CDK1 and CCNB1 in liver cancer. Oncol Lett. 2021;22:653. 10.3892/ol.2021.12914. 34386075 PMC8299009

[68] Komatsu S, Takenobu H, Ozaki T et al. Plk1 regulates liver tumor cell death by phosphorylation of TAp63. Oncogene. 2009;28:3631–41. 10.1038/onc.2009.216. 19668228

[69] Zheng Y, Shi Y, Yu S et al. GTSE1, CDC20, PCNA, and MCM6 synergistically affect regulations in cell cycle and indicate poor prognosis in liver cancer. Anal Cell Pathol. 2019;2019:1038069. 10.1155/2019/1038069. PMC7012210 32082966

[70] Zhang L, Zhang Y, Wang C et al. Integrated single-cell RNA sequencing analysis reveals distinct cellular and transcriptional modules associated with survival in lung cancer. Signal Transduct Target Ther. 2022;7:9. 10.1038/s41392-021-00824-9. 35027529 PMC8758688

[71] Wang D, Wan B, Zhang X et al. Nuclear respiratory factor 1 promotes the growth of liver hepatocellular carcinoma cells via E2F1 transcriptional activation. BMC Gastroenterol. 2022;22:198. 10.1186/s12876-022-02260-7. 35448958 PMC9027447

[72] Sun L, Liu T, Zhang S et al. Oct4 induces EMT through LEF1/β-catenin dependent WNT signaling pathway in hepatocellular carcinoma. Oncol Lett. 2017;13:2599–606. 10.3892/ol.2017.5788. 28454439 PMC5403449

[73] Xu Y, Zhang X, Zhang R et al. AFP deletion leads to anti-tumorigenic but pro-metastatic roles in liver cancers with concomitant CTNNB1 mutations. Cancer Lett. 2023;566:216240. 10.1016/j.canlet.2023.216240. 37217071

[74] Pasche B, Pennison MJ, Jimenez H et al. TGFBR1 and cancer susceptibility. Trana Am Clin Climatol Assoc. 2014;1:300. PMC4112675 25125747

[75] SuáKim K, WooáHong S, HyunáYun S et al. Hyaluronate–Flt1 peptide conjugate/epirubicin micelles for theranostic application to liver cancers. Rsc Adv. 2015;5:48615–8. 10.1039/C5RA07464A.

[76] Nie K, Li J, Peng L et al. Pan-cancer analysis of the characteristics of LY96 in prognosis and immunotherapy across human cancer. Front Mol Biosci. 2022;9:837393. 10.3389/fmolb.2022.837393. 35647025 PMC9130738

[77] Qi Y, Xu F, Chen L et al. Quantitative proteomics reveals FLNC as a potential progression marker for the development of hepatocellular carcinoma. Oncotarget. 2016;7:68242. 10.18632/oncotarget.11921. 27626164 PMC5354476

[78] Lin F, Yang H, Huang Z et al. Magnesium-related gene ITGAL: a key immunotherapy predictor and prognostic biomarker in pan-cancer. Front Pharmacol. 2024;15:1464830. 10.3389/fphar.2024.1464830. 39605903 PMC11598444

[79] Dixon DA, Tolley ND, King PH et al. Altered expression of the mRNA stability factor HuR promotes cyclooxygenase-2 expression in colon cancer cells. J Clin Invest. 2001;108:1657–65. 10.1172/JCI12973. 11733561 PMC200983

[80] Fernau NS, Fugmann D, Leyendecker M et al. Role of HuR and p38MAPK in ultraviolet B-induced post-transcriptional regulation of COX-2 expression in the human keratinocyte cell line HaCaT. J Biol Chem. 2010;285:3896–904. 10.1074/jbc.M109.081430. 19917608 PMC2823532

[81] Aloufi N, Haidar Z, Ding J et al. Role of human antigen R (HuR) in the regulation of pulmonary ACE2 expression. Cells. 2021;11:22. 10.3390/cells11010022. 35011584 PMC8750694

[82] Chang SH, Lu YC, Li X et al. Antagonistic function of the RNA-binding protein HuR and miR-200b in post-transcriptional regulation of vascular endothelial growth factor-A expression and angiogenesis. J Biol Chem. 2013;288:4908–21. 10.1074/jbc.M112.423871. 23223443 PMC3576095

[83] Yiakouvaki A, Dimitriou M, Karakasiliotis I et al. Myeloid cell expression of the RNA-binding protein HuR protects mice from pathologic inflammation and colorectal carcinogenesis. J Clin Invest. 2012;122:48–61. 10.1172/JCI45021. 22201685 PMC3248801

[84] Al-Habeeb F, Aloufi N, Traboulsi H et al. Human antigen R promotes lung fibroblast differentiation to myofibroblasts and increases extracellular matrix production. J Cell Physiol. 2021;236:6836–51. 10.1002/jcp.30380. 33855709 PMC8390431

[85] Trivlidis J, Aloufi N, Al-Habeeb F et al. HuR drives lung fibroblast differentiation but not metabolic reprogramming in response to TGF-β and hypoxia. Resp Res. 2021;2:1–19. 10.1186/s12931-021-01916-4. PMC8715577 34963461

[86] Patil M, Singh S, Dubey PK et al. Fibroblast-specific depletion of human antigen R alleviates myocardial fibrosis induced by cardiac stress. JACC Basic Transl Sci. 2024;9:754–70. 10.1016/j.jacbts.2024.03.004. 39070272 PMC11282885

[87] Zhao B, Su T, Wang J et al. DENetwork unveils non-differentially expressed genes with functional relevance across conditions through information flow perturbation. Zenodo, 2025. 10.5281/zenodo.17211767. PMC12709196 41404797

[88] Zhao B, Su T, Wang J et al. DENetwork unveils non-differentially expressed genes with functional relevance across conditions through information flow perturbation. Zenodo 2025. 10.5281/zenodo.17211695. PMC12709196 41404797

