# Supplementary material for: DENetwork unveils non-differentially expressed genes with functional relevance across conditions through information flow perturbation
Source: Nucleic Acids Res. 2025 Dec 17;53(22):gkaf1350. doi: 10.1093/nar/gkaf1350 (PMC12709196; doi:10.1093/nar/gkaf1350)
Supplement: gkaf1350_Supplemental_Files [file gkaf1350_supplemental_files.zip › DENetwork_Supplementary.pdf]

Supplementary Figures

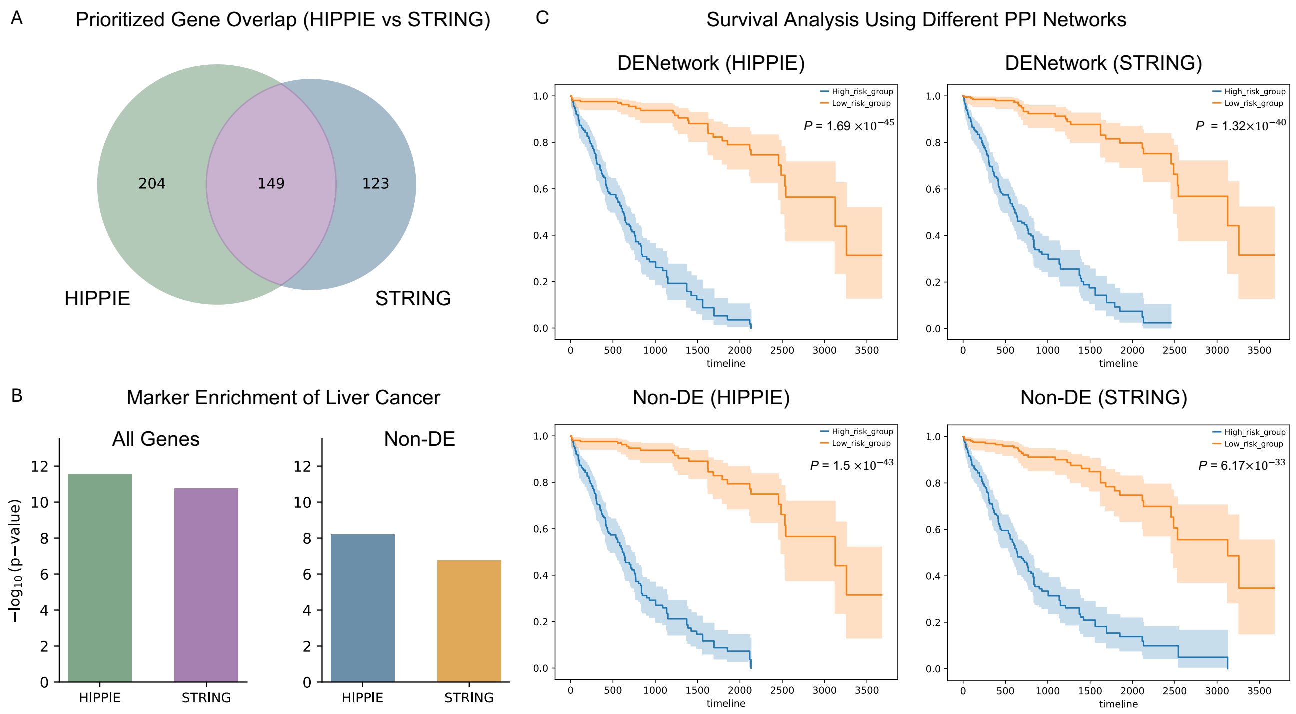

Supplementary Figure S1: Comparison of DENetwork results using HIPPIE and STRING as PPI inputs. **(A)** Venn diagram of prioritized genes identified by DENetwork using HIPPIE and STRING as inputs. **(B)** Hypergeometric enrichment analysis of prioritized genes against curated liver cancer marker sets, shown for all genes and non-DE genes. **(C)** Kaplan–Meier survival analysis comparing high-risk and low-risk patient groups stratified by DENetwork-prioritized genes, shown for all genes and non-DE genes under HIPPIE and STRING inputs.

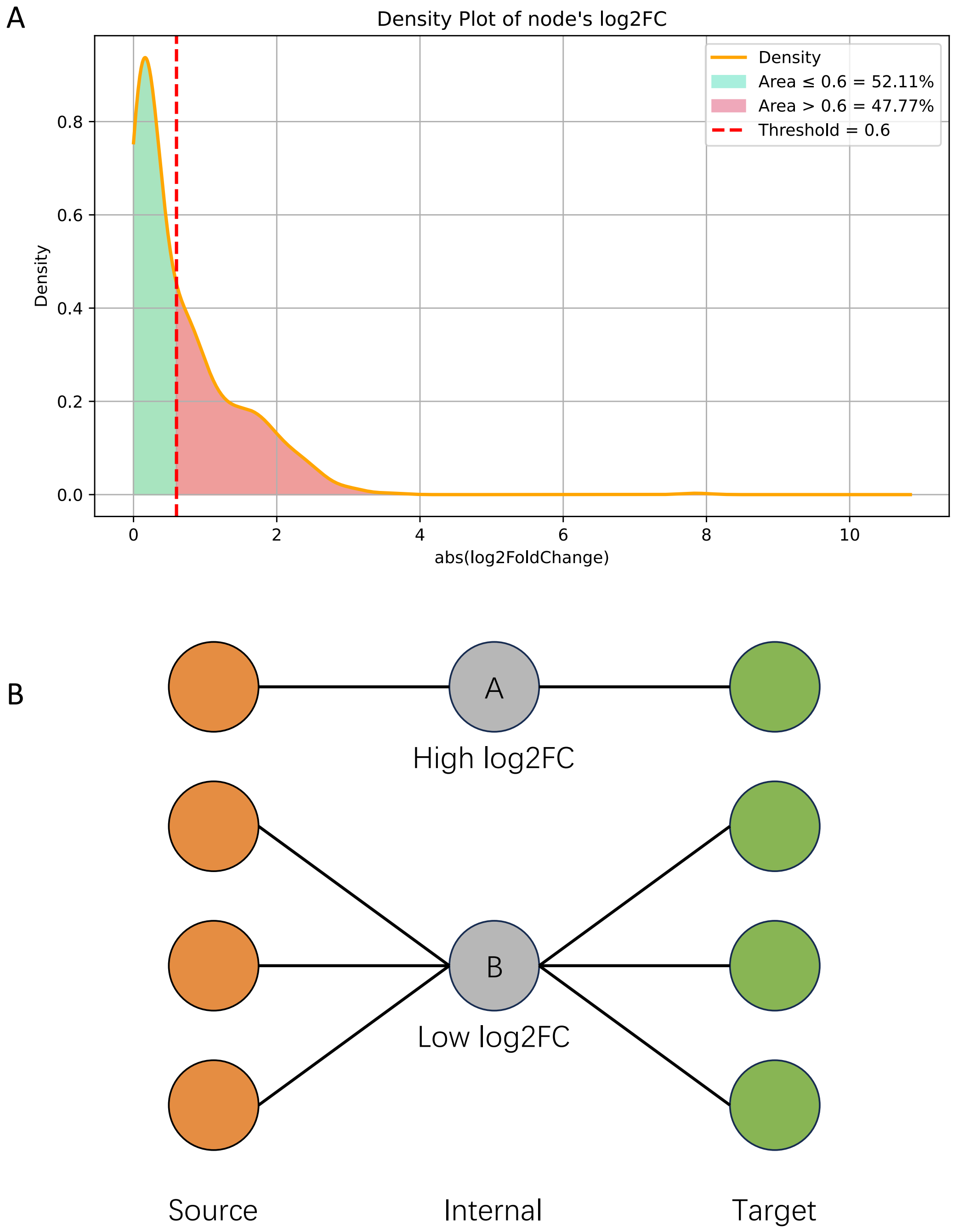

Supplementary Figure S2: DENetwork prioritizes genes based on network signal flow rather than expression magnitude alone. **(A)** Density plot of the $|\log_2(\text{fc})|$ values for all nodes included in the final optimal local network for the Macrophage dataset. The vertical red dashed line marks the threshold of $\log_2(\text{fc}) = 0.6$. The green and red shaded areas represent the proportions of nodes with $\log_2(\text{fc}) \leq 0.6$ (52.11%) and $\log_2(\text{fc}) > 0.6$ (47.77%), respectively, indicating that a substantial number of prioritized genes exhibit relatively small expression changes. **(B)** Schematic diagram comparing two representative internal nodes: Node A (high $|\log_2(\text{fc})|$), low connectivity and Node B (low $|\log_2(\text{fc})|$, high connectivity). Source nodes are shown in orange, internal nodes in gray, and target nodes in green. This example illustrates that DENetwork prioritizes nodes based on the amount of signal flow they mediate within the network, rather than solely on transcriptional magnitude. Consequently, genes with modest expression changes can still be ranked highly if they function as essential conduits in signal propagation.

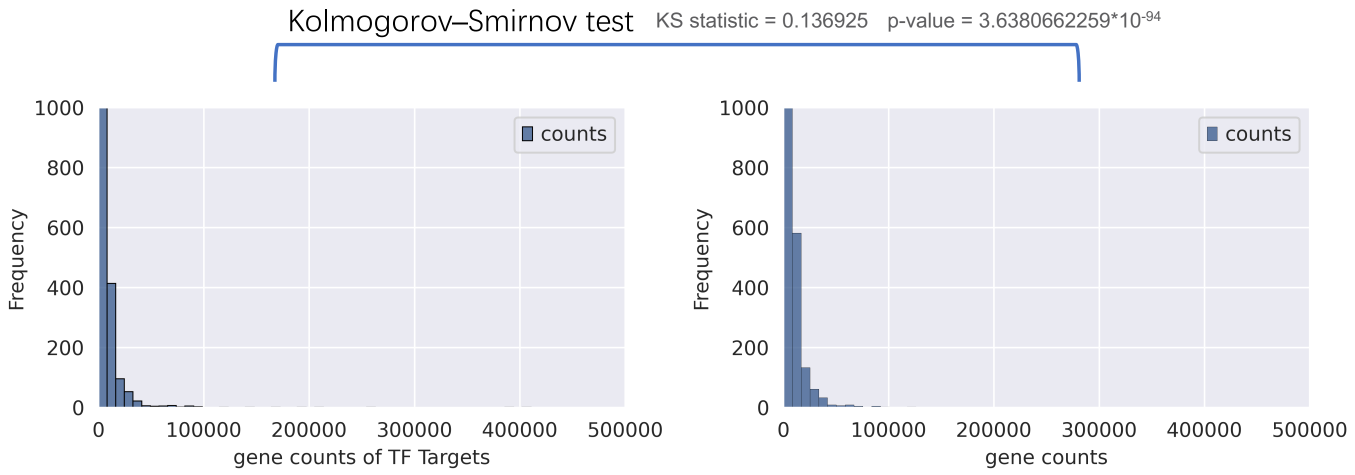

Supplementary Figure S3: Comparison of expression count distributions between TF target genes and all genes in the Influenza dataset. Histograms show the distribution of total gene expression counts across all samples for TF target genes (left) and all genes (right). A Kolmogorov–Smirnov (KS) test was performed to evaluate the difference between the two distributions, yielding a KS statistic of 0.137 and a p-value of 3.64×10^{-94}.

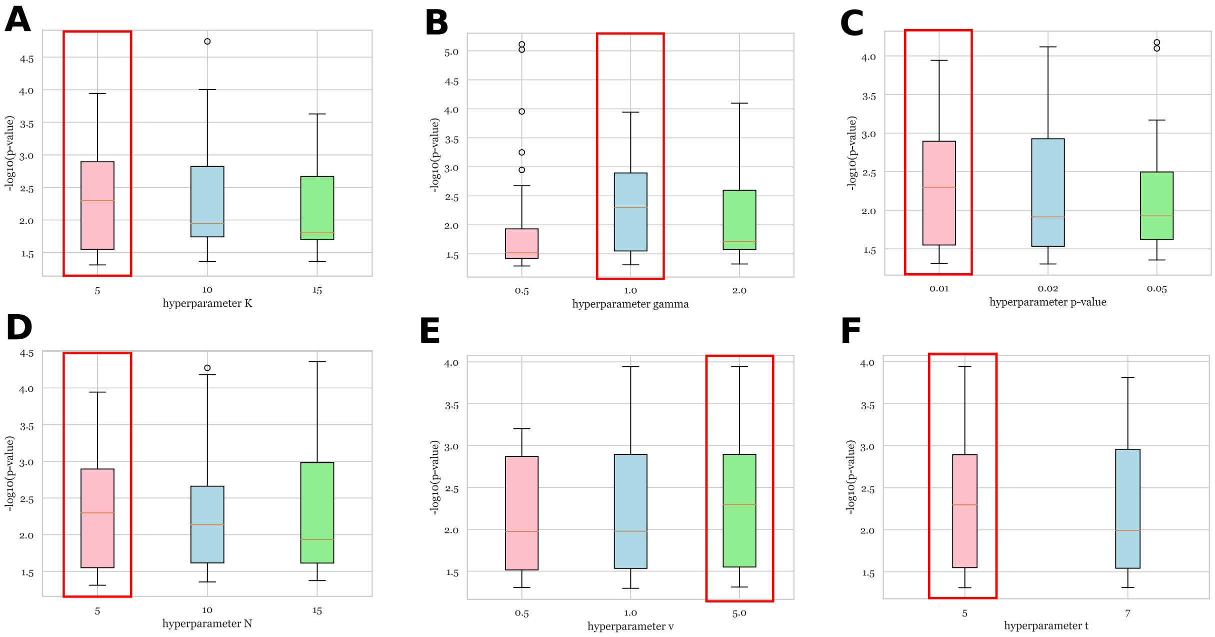

Supplementary Figure S4: Hyperparameter tuning of DENetwork. Boxplots showing the effect of different hyperparameter settings on Reactome pathway enrichment across five benchmark datasets. Median $-\log_{10}(P)$ values are indicated by orange lines, and the selected parameters are boxed in red. **(A)** Maximum number of internal nodes ($K = 5$). **(B)** Regularization coefficient ($\gamma = 1$). **(C)** Pathway significance threshold ($p = 0.01$). **(D)** Number of random permutations ($N = 5$). **(E)** Edge retention threshold ($v = 5\%$). **(F)** Iteration number ($t = 5$).

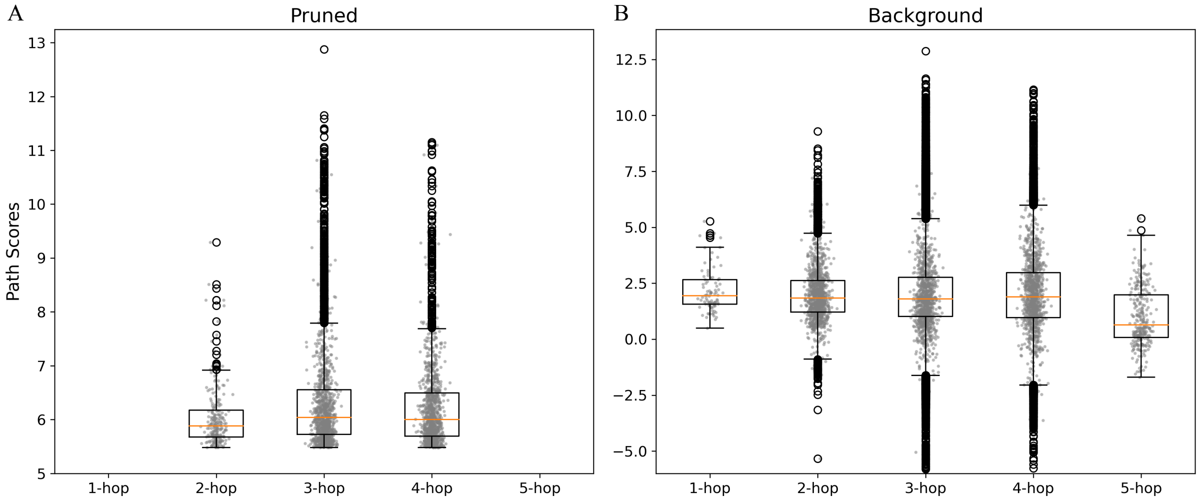

Supplementary Figure S5: Distribution of DENetwork path scores in the HuR dataset. (A) Significant paths retained after permutation-based pruning. **(B)** Background distributions of all enumerated paths from 1 to 5 hops.

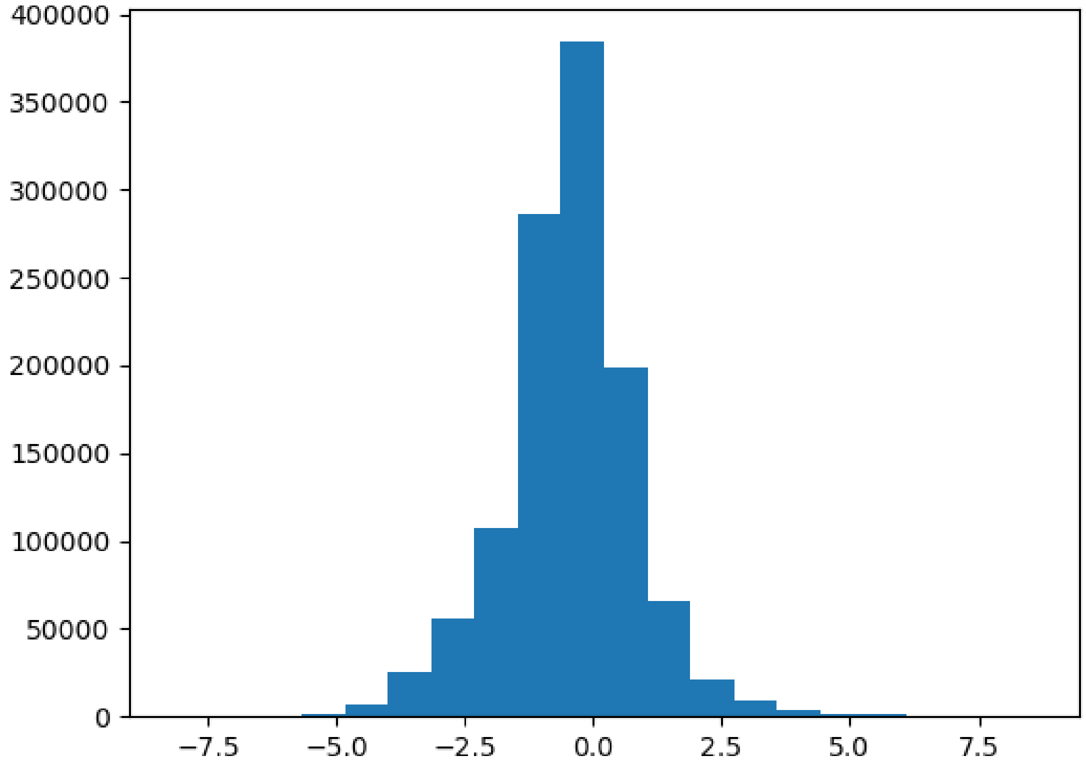

Supplementary Figure S6: The path score distribution. Number of paths is plotted against path scores for the initial fully connected network of the Influenza dataset, showing an approximately Gaussian distribution.

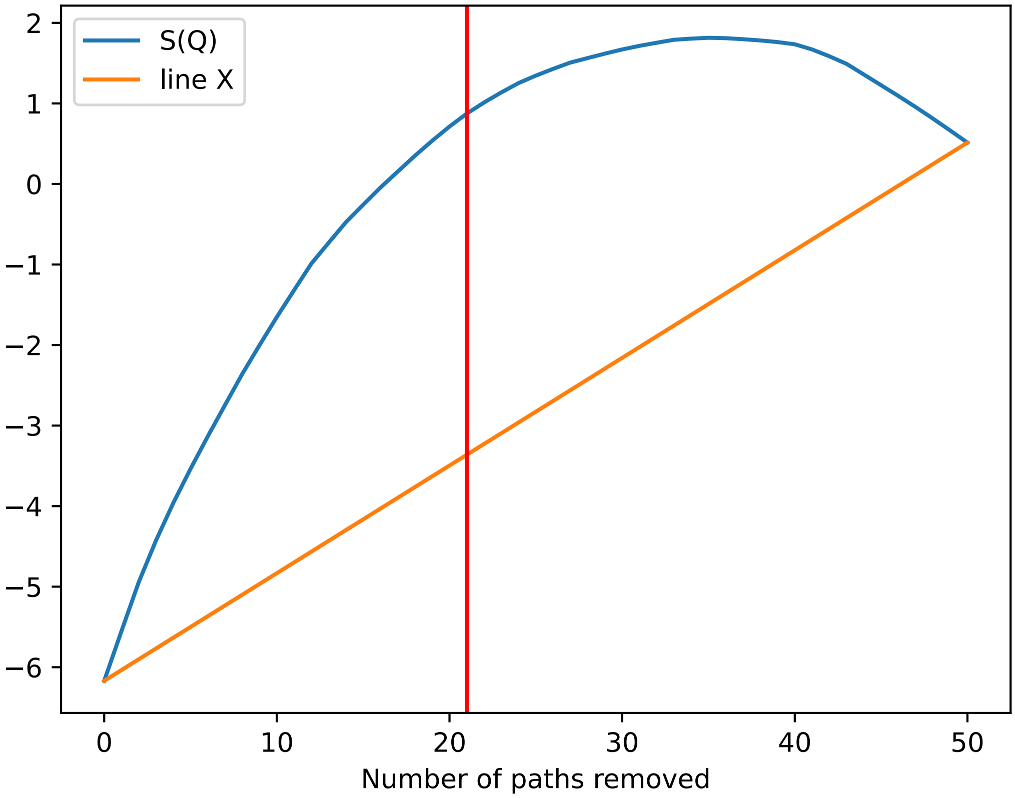

Supplementary Figure S7: Identification of the knee point in the IAV dataset. The plot shows $S(Q)$ (blue) against the number of paths removed (i). The knee point, occurring when 21 paths are removed, is highlighted in red. The orange line (X) connects the first point (no paths removed) and the last point (50 paths removed). Among multiple possible knees, the one with the largest Euclidean distance to line X is selected to define the local-optimal network.

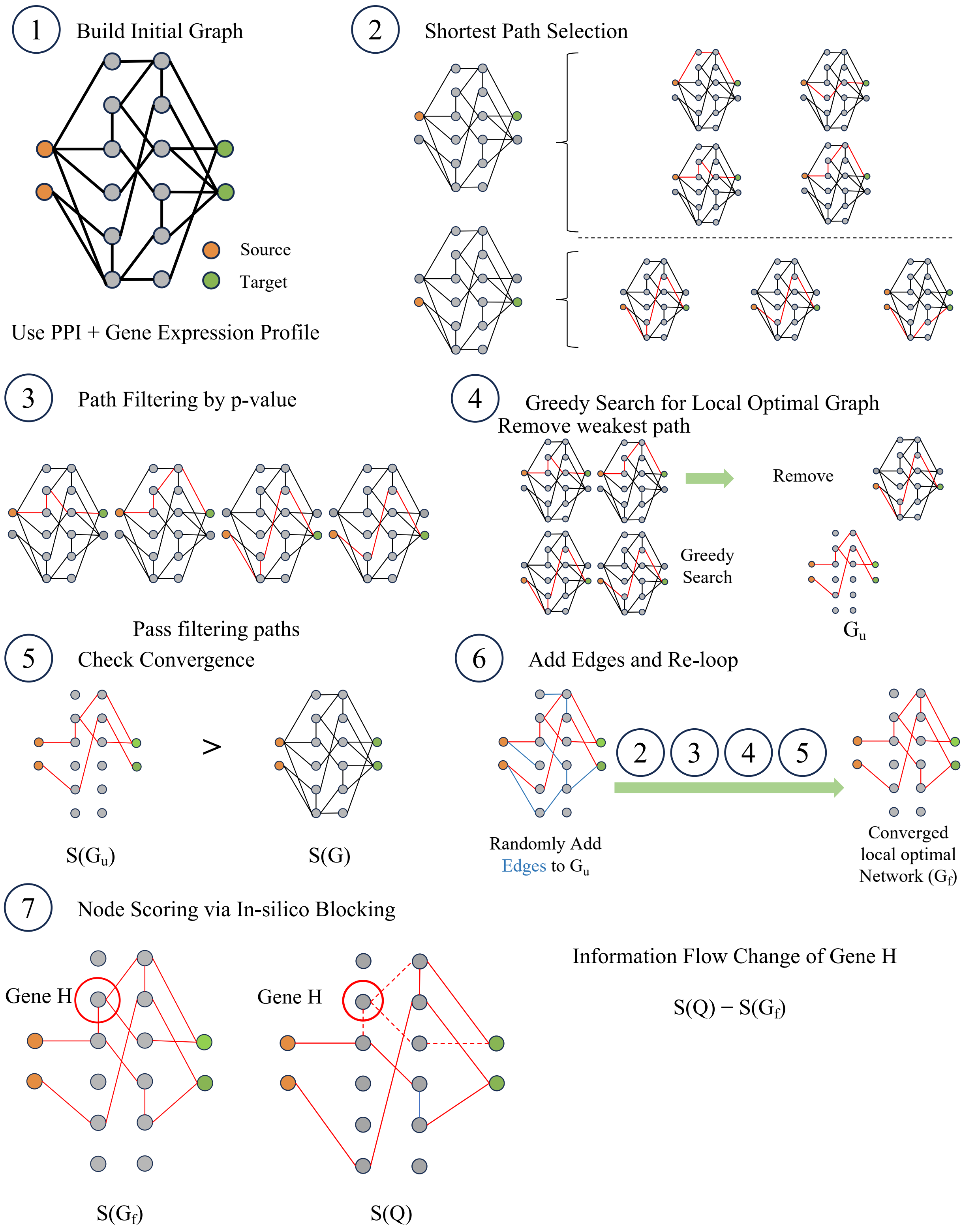

Supplementary Figure S8: Step-by-step local optimal graph refinement in DENetwork.

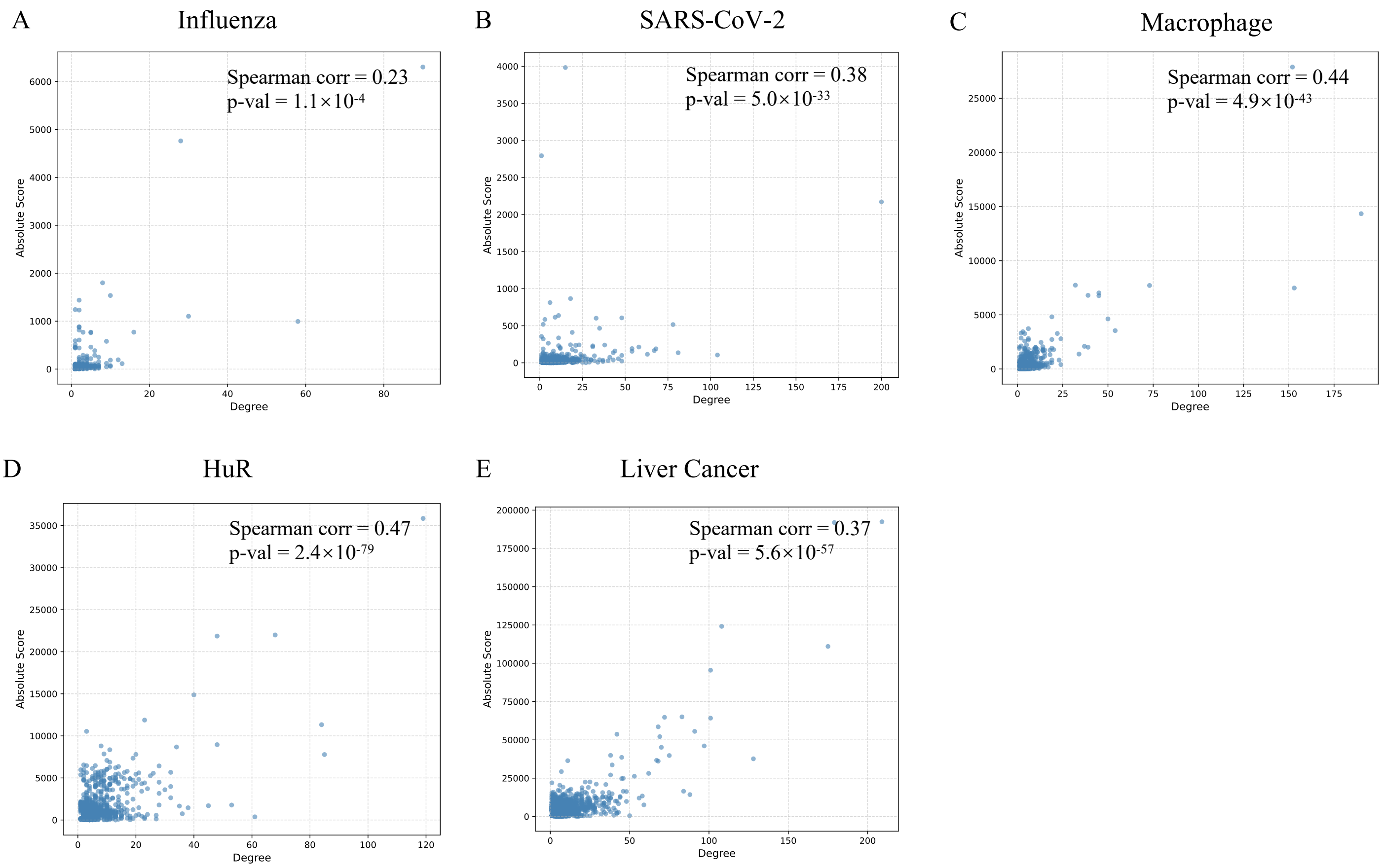

Supplementary Figure S9: Relationship between node degree and importance score across datasets. Scatter plots showing the relationship between node degree (x-axis) and DENetwork importance score (y-axis) for five datasets: **(A)** Influenza, **(B)** SARS-CoV-2, **(C)** Macrophage (*Alox15*$^{-/-}$), **(D)** HuR knockdown, and **(E)** Liver cancer. Each dot represents a gene. Spearman correlation coefficients and corresponding p-values are shown in each panel.

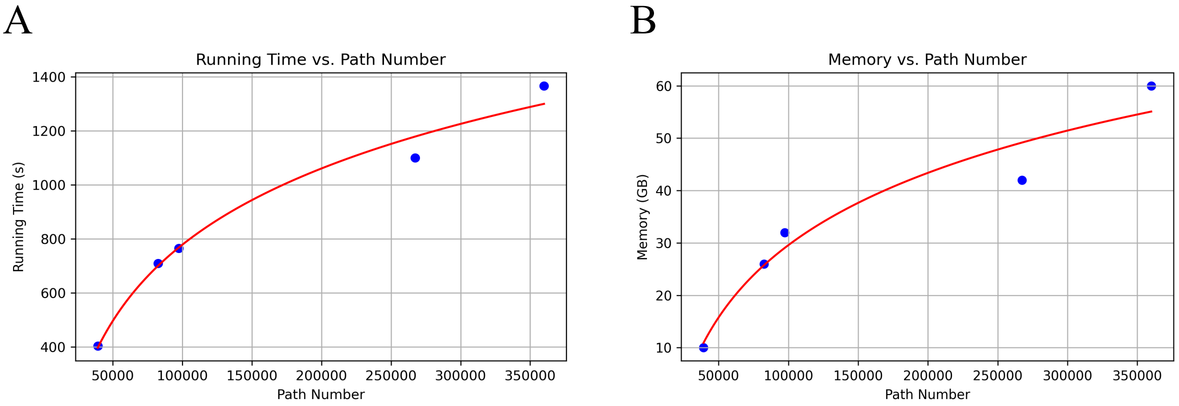

Supplementary Figure S10: Computational cost of DENetwork as a function of path number. (A) Runtime (seconds) and **(B)** peak memory usage (GB) are plotted against the total number of paths in the initial graph. Different path counts were generated by randomly down-sampling the input gene set from the Influenza dataset. Each dot represents one DENetwork run on a sub-sampled network, and the red curve shows the best-fit logarithmic trend.

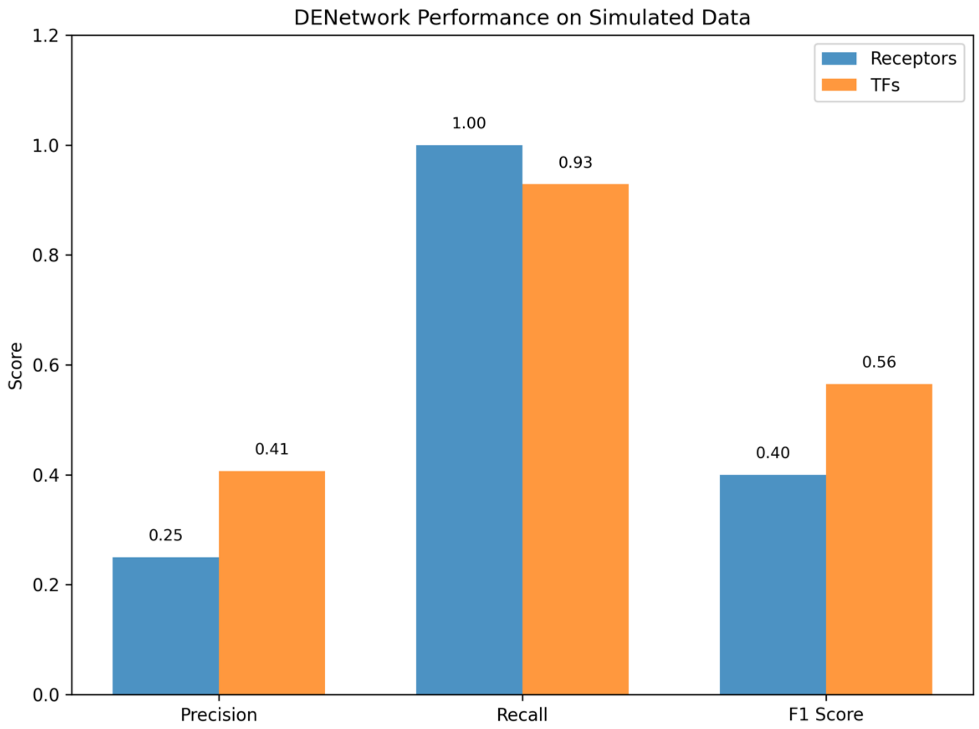

Supplementary Figure S11: Performance of DENetwork in recovering implanted regulators from simulated data. Bar plot showing the precision, recall, and F1 score for identifying implanted receptors and transcription factors (TFs) using the DENetwork method. All 8 implanted receptors and 13 out of 14 TFs were correctly identified among the top 100 ranked nodes. While recall was high for both receptors (1.0) and TFs (0.93), the precision was lower (0.25 for receptors and 0.41 for TFs), resulting in F1 scores of 0.40 and 0.56, respectively.

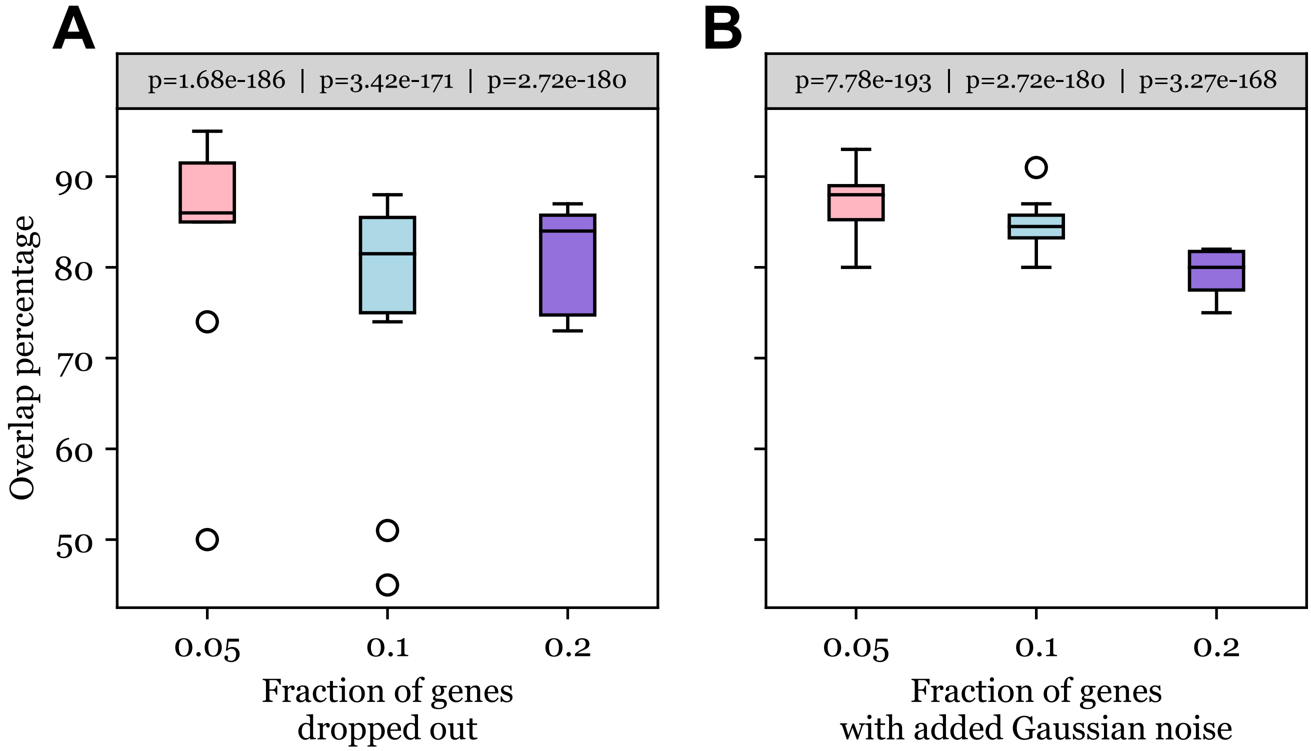

Supplementary Figure S12: DENetwork is robust against transcriptomic noise. The boxplots represent the percent conservation of the top 100 genes within the DENetwork when subject to varying levels of simulated noise on the IAV dataset. Two prevalent types of noise found in transcriptomic measurements were simulated for this test. Panel **(A)** illustrates the resilience of the DENetwork against dropout noise, and Panel **(B)** demonstrates its robustness when exposed to Gaussian noise. The x-axis in both panels indicates the level of noise, while the y-axis signifies the robustness of the network, represented by the percent conservation of the top 100 genes under noise simulation (relative to the results without simulated noise). Noise was simulated within 5% (pink), 10% (blue), and 20% (purple) of the genes. Statistical significance of gene overlap under noise simulation, reflected by the p-value, is displayed above each corresponding box.

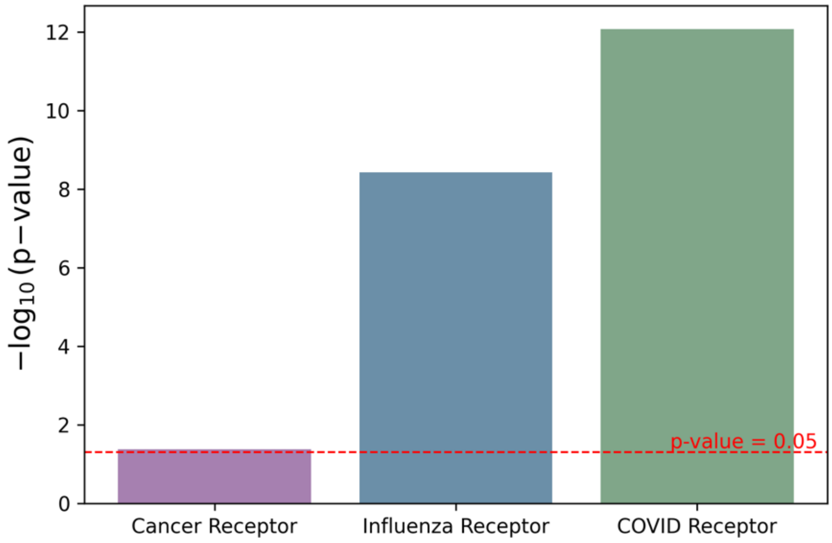

Supplementary Figure S13: Hypergeometric enrichment analysis of disease-associated genes for selected receptors. The plot shows the statistical significance of enrichment (-$\log_{10}$(p-value)) for genes associated with Cancer, Influenza, and SARS-CoV-2, based on known disease gene sets from publicly available databases.

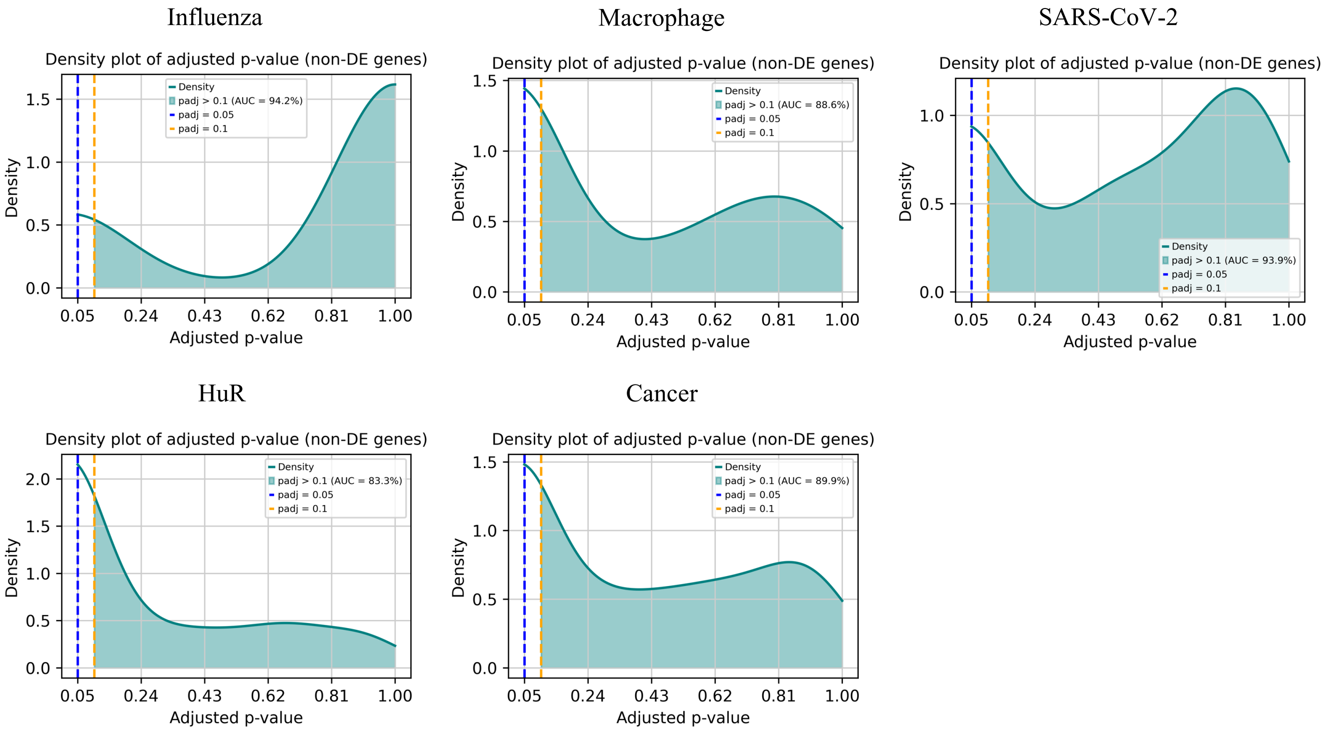

Supplementary Figure S14: Density plots of adjusted p-values (padj) for genes prioritized by DENetwork as non-differentially expressed (non-DE) across five datasets. These genes were excluded from the set of DE genes based on conventional differential expression criteria ($|\log_2(\text{fc})| = 0.6$) and $P \leq 0.05$) but were identified by DENetwork as potentially functionally important. The shaded regions indicate the proportion of these DENetwork-prioritized non-DE genes with $P > 0.1$, with the corresponding area under the curve (AUC) reported in the legend. Vertical dashed lines mark the significance thresholds at $P = 0.05$ (blue) and $P = 0.1$ (orange). Each panel represents one dataset: Influenza, Macrophage, SARS-CoV-2, HuR knockdown, and Liver Cancer. These results demonstrate that most DENetwork-prioritized non-DE genes maintain adjusted p-values well above traditional significance cutoffs, supporting their classification as genuinely non-differentially expressed under standard statistical criteria.

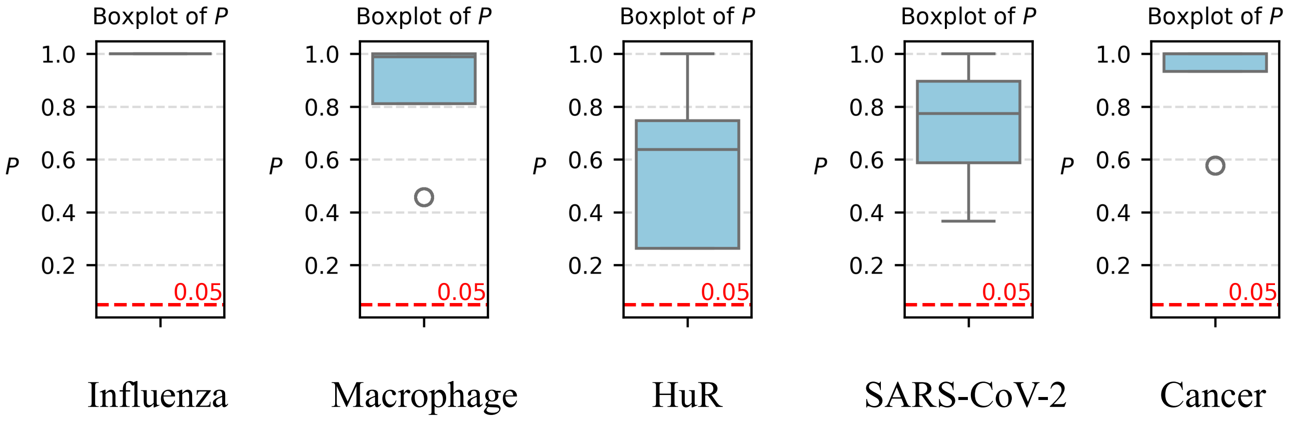

Supplementary Figure S15: Boxplots showing the distribution of FDR-adjusted *P* values obtained from the two-sided Wilcoxon rank-sum test for each dataset. The red dashed line indicates the commonly used significance threshold ($P = 0.05$). Results are shown for five datasets: Influenza, Macrophage, HuR, SARS-CoV-2, and Cancer.

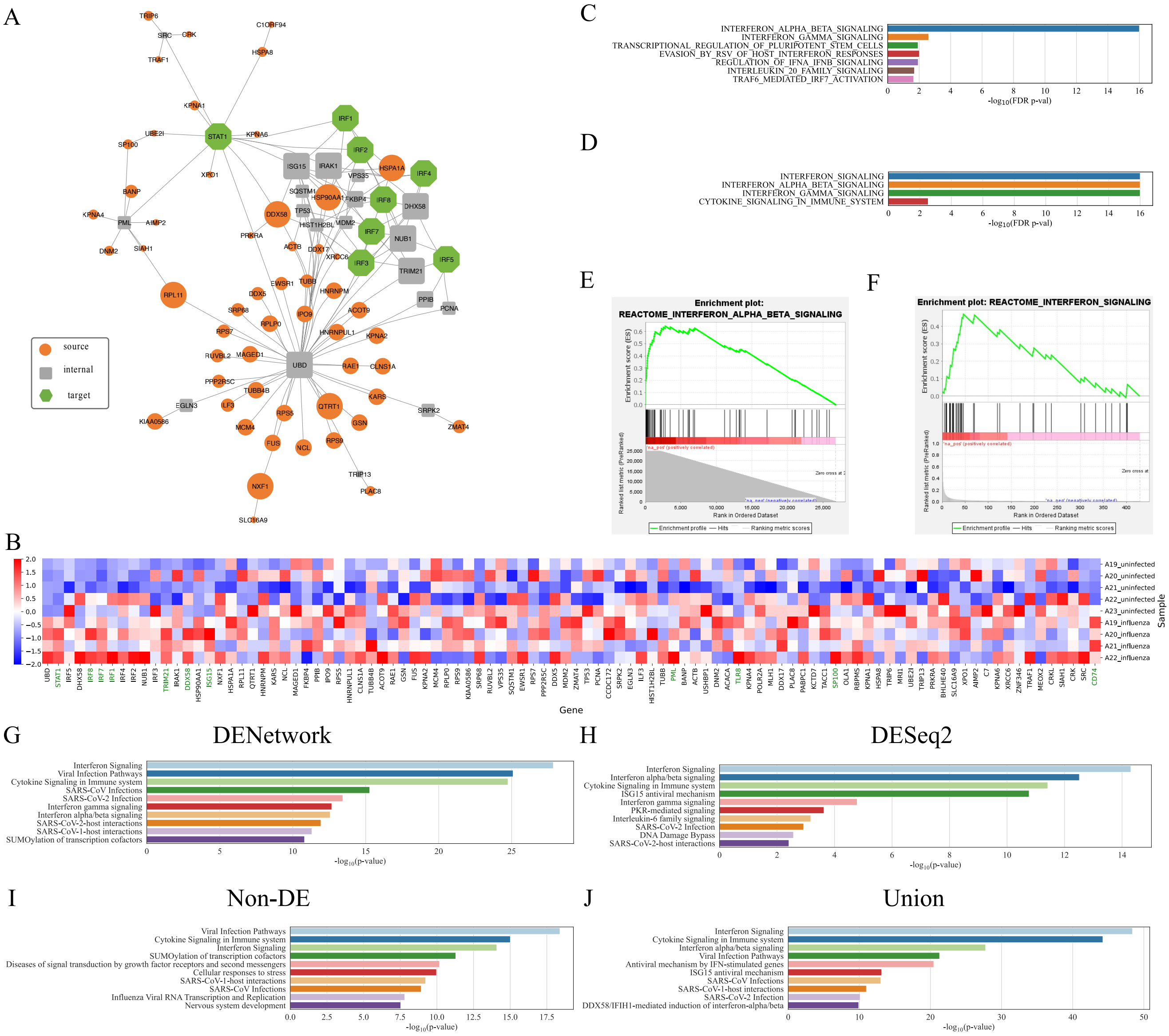

Supplementary Figure S16: Transcription factor (TF) module analysis reveals key regulatory factors in the influenza dataset. **(A)** Network visualization of the top 100 genes prioritized by DENetwork, with source nodes (orange), internal nodes (gray), and target nodes (green). Node size indicates network influence. **(B)** Heatmap of z-score normalized expression for the top 100 DENetwork genes across control and influenza-infected samples. DE genes are labeled in green, non-DE genes in black. **(C, D)** Reactome pathway enrichment analysis for DENetwork-ranked genes (C) and DESeq2-ranked DE genes (D), highlighting both shared and unique immune-related pathways. **(E, F)** GSEA enrichment plots of representative pathways identified by DENetwork (E) and DESeq2 (F) rankings. **(G–J)** Reactome pathway enrichment results for four gene sets: (G) all DENetwork genes, (H) DESeq2-identified DE genes, (I) DENetwork-specific non-DE genes, and (J) the union of DENetwork and DESeq2 gene sets. Top 15 pathways are shown for each panel.

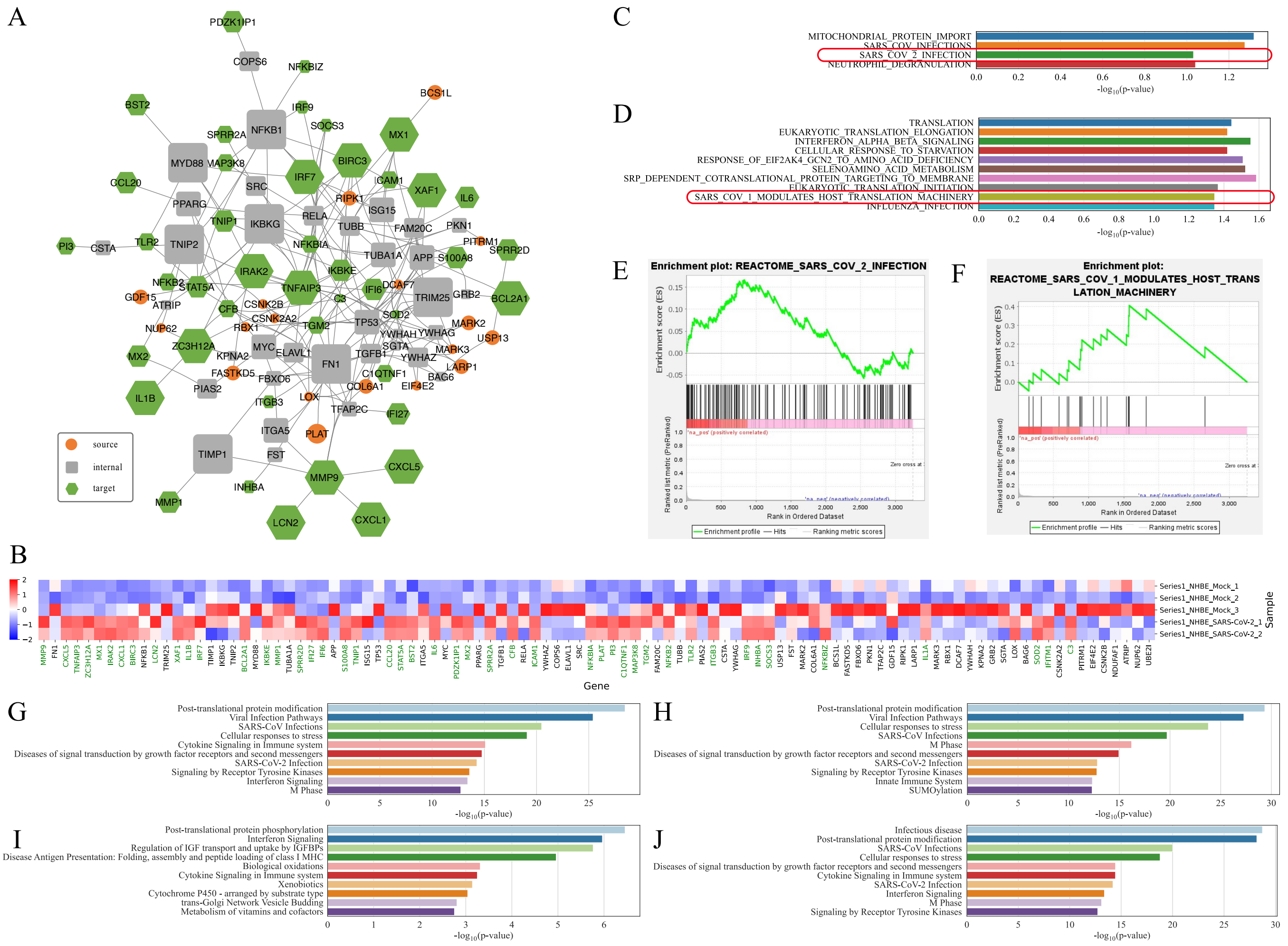

Supplementary Figure S17: DENetwork identifies crucial DE and non-DE genes associated with SARS-CoV-2 infections. **(A)** Network visualization of the top 100 genes prioritized by DENetwork, with source nodes (orange), internal nodes (gray), and target nodes (green). Node size indicates network influence. **(B)** Heatmap of z-score normalized expression for the top 100 DENetwork genes across SARS-CoV-2 positive and negative samples. Upregulated genes are labeled in green. **(C, D)** GSEAPreRanked pathway enrichment analysis for the DENetwork gene set ranked by DENetwork scores (C) and by DESeq2 FDR-adjusted p-values (D). Top pathways are highlighted in red. **(E, F)** GSEA enrichment plots of representative pathways identified by DENetwork (E) and DESeq2 (F) rankings. **(G–J)** Reactome pathway enrichment results for four gene sets: (G) all DENetwork genes, (H) DENetwork-specific non-DE genes, (I) DESeq2-identified upregulated genes, and (J) the union of DENetwork and DESeq2 gene sets. Top pathways are shown for each panel.

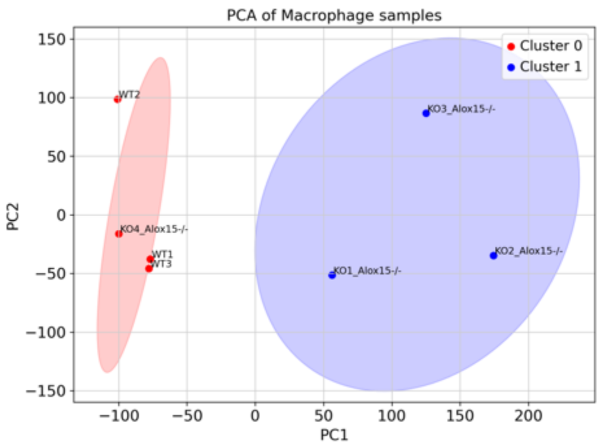

Supplementary Figure S18: Principal Component Analysis (PCA) of macrophage samples. PCA was performed based on gene expression profiles to assess sample clustering. Samples are colored by cluster assignments: Cluster 0 (red) and Cluster 1 (blue).

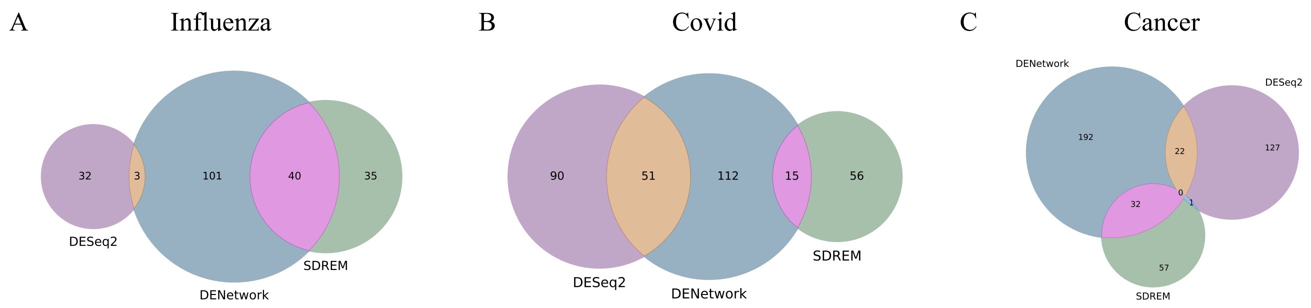

Supplementary Figure S19: Venn diagrams showing the overlap of genes identified by DENetwork, SDREM, and DESeq2 in three datasets. **(A)** Influenza dataset. **(B)** SARS-CoV-2 dataset. **(C)** Liver cancer dataset.
